# Ribosome reinitiation at leader peptides increases translation of bacterial proteins

**DOI:** 10.1186/s13062-016-0123-8

**Published:** 2016-04-16

**Authors:** Semen A. Korolev, Oleg A. Zverkov, Alexandr V. Seliverstov, Vassily A. Lyubetsky

**Affiliations:** Institute for Information Transmission Problems of the Russian Academy of Sciences (Kharkevich Institute), Bolshoy Karetny per. 19, build.1, Moscow, 127051 Russia

**Keywords:** Bacteria, Leader gene, Ribosome, Leader gene-structural gene distance, Reinitiation

## Abstract

Short leader genes usually do not encode stable proteins, although their importance in expression control of bacterial genomes is widely accepted. Such genes are often involved in the control of attenuation regulation. However, the abundance of leader genes suggests that their role in bacteria is not limited to regulation. Specifically, we hypothesize that leader genes increase the expression of protein-coding (structural) genes via ribosome reinitiation at the leader peptide in the case of a short distance between the stop codon of the leader gene and the start codon of the structural gene. For instance, in Actinobacteria, the frequency of leader genes at a distance of 10–11 bp is about 70 % higher than the mean frequency within the 1 to 65 bp range; and it gradually decreases as the range grows longer. A pronounced peak of this frequency-distance relationship is also observed in Proteobacteria, Bacteroidetes, Spirochaetales, Acidobacteria, the Deinococcus-Thermus group, and Planctomycetes. In contrast, this peak falls to the distance of 15–16 bp and is not very pronounced in Firmicutes; and no such peak is observed in cyanobacteria and tenericutes. Generally, this peak is typical for many bacteria. Some leader genes located close to a structural gene probably play a regulatory role as well.

**Reviewers:** This article was reviewed by Piotr Zielenkiewicz and István Simon.

**Electronic supplementary material:**

The online version of this article (doi:10.1186/s13062-016-0123-8) contains supplementary material, which is available to authorized users.

## Findings

### Background

A large-scale study of leader genes has been carried out in bacteria. Usually, leader genes are short open reading frames not specified in genome annotations; they encode no stable proteins of independent significance. Leader genes play an important role in the regulation of gene expression in bacteria; this role with regard to Rho protein was described in [1, 2]. Basically, leader genes are involved in the attenuation mechanism, which relies on coupling of transcription and translation; specifically, on the dependence of the elongation rate by the ribosome on the concentration of specific amino acids and aminoacyl-tRNA synthetases. Such regulation has been first described in *Escherichia coli* [3]; it is also known in Actinobacteria. Experimental support for the tryptophan concentration-dependent regulation was obtained for *Corynebacterium glutamicum* [4] and *Streptomyces venezuelae* [5]. Large-scale bioinformatic searches for this regulation has been carried out elsewhere [1, 2, 6]. A further search and an expansion of the initial notion of the attenuation mechanism can be found in [7]. In particular, the importance of the chains of RNA helices, RNA triplexes, environmental temperature, the mechanism relying on overlapping the ribosome binding site, etc. was outlined in this work. For instance, a computer model of the attenuation mechanism [6] cannot reveal the dependence of *trpE* gene expression on tryptophanyl-tRNA concentration in *S. venezuelae* without considering RNA triplexes, while this relationship becomes apparent when RNA triplexes are taken into account [8], which agrees with experimental data [5]. Another example, RNA triplexes with predicted critical role in the regulation depending on histidyl-tRNA were found upstream of the *hisG* gene in many gamma-proteobacteria including representatives of the Enterobacteriales, Pasteurellales, Vibrionales, Alteromonadales, and Aeromonadales families; upstream of the *hisZ* gene in *Clostridium difficile*, *Bacillus cereus*, *B. thuringiensis*, *B. Anthracis*, and *B. weihenstephanensis*; as well as upstream of the *lysQ* gene in *Lactococcus lactis*, the product of which, permease, has altered substrate specificity relative to putative orthologs [7].

Leader genes play a critical role in gene expression not only in prokaryotes but also in certain protozoans including trypanosomes. In this case, a short RNA with a 5'-cap is joined to an independently transcribed protein-coding RNA as a result of trans-splicing [9]. Long RNAs initially lacking the 5'-cap can be processed from a polycistronic transcript.

Eubacterial ribosome covers about 12 nt of the RNA downstream of the translated codon [10] (a brief review of ribosome structure can be found in [6]). This defines the upper limit of the distance between genes that allows ribosome reinitiation.

Hereafter, protein-coding genes annotated in the NCBI database are referred to as structural. If the stop codon of a leader gene is immediately adjacent to the start codon of a structural gene, the distance between them is taken equal to 1. The leader genes predicted here do not overlap structural genes on either DNA strand and their mean length is small. The analyzed genomes are listed in the Additional file 1; the mean length of leader genes can be found in Additional file 2, part 1; while the bulky tables of identified leader-structural gene pairs can be downloaded from [11].

We assume that the role of leader genes in bacteria is not limited to the attenuation and similar regulatory mechanisms even allowing for the variants proposed in [7]. In the case of a short distance between the stop codon of a leader gene and start codon of the structural gene, these genes constitute a common operon and the former gene increases the rate of translation initiation of the latter one. In addition, some of such leader genes probably play a regulatory role.

### Methods

Bacterial genomes were retrieved from GenBank. Gene annotations were verified using the Pfam database [12]. Leader genes were identified using the method described in [2]. The longest open reading frame in the 5'-leader of a structural gene not overlapped with other structural genes on both DNA strands was considered as a leader gene. Thus, each structural gene could have no more than one leader gene; the corresponding list is available at [11]; and the results were obtained using this set of gene pairs.

The sequence logos were generated using the WebLogo tool [13]. RNA secondary structures were predicted using RNAstructure [14].

### Results

Bacteria demonstrate an unusually high frequency of the 10–11 bp distance between the leader and structural genes. A less pronounced local peak is observed for the 5 bp distance.

**Fig. 1:**
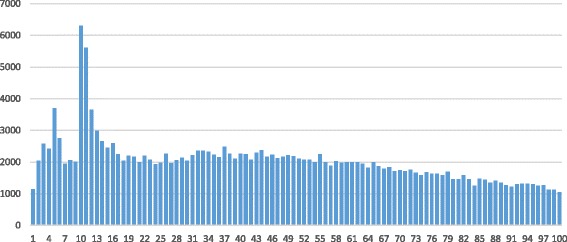
The number of predicted leader genes as a function of the distance between the leader and structural genes in Actinobacteria

Figure 1 shows the number of predicted leader genes in Actinobacteria as a function of the distance between the leader and structural genes. The frequency of leader genes at a distance of 10–11 bp is about 70 % higher than the mean frequency within the 1 to 65 bp range; and it gradually decreases as the range grows longer. Similar peaks for the distance of 10–11 bp are observed in certain actinobacterial genera with a large number of species: *Corynebacterium*, *Mycobacterium*, and *Streptomyces*. Minor peaks for the 3, 5–6, 10–12, and 37 bp distances between the leader and structural genes are observed in the early diverged genus *Bifidobacterium*.

All these results are summarized in the diagrams in Additional file 2, part 2.

A pronounced peak is observed in Proteobacteria as a whole as well as in individual taxonomic groups. In alpha-, beta-, epsilon-, delta-, and gamma-proteobacteria, the distance of 10–13 bp peaks; although it is less pronounced in gamma-proteobacteria; while epsilon-proteobacteria demonstrate a sharp peak for the distance of 10–11 bp. In Bacteroidetes, the distance of 11–14 bp peaks.

The peaks of leader gene frequencies at these distances are observed in Spirochaetales, Acidobacteria, the Deinococcus-Thermus group, and Planctomycetes. Notably, the peak in Planctomycetes becomes more pronounced after the genus *Planctomyces* is excluded.

On the contrary, the peak in Firmicutes corresponds to the distance of 15–16 bp and is not very pronounced. The considered peak is absent in cyanobacteria and tenericutes, which remains true after filtering out genera with many species as indicated in the Additional file 1. Specifically, the filtering consisted in selecting a single genome from each genus; the filtered list of genomes in the Additional file 1 is marked as “one genome per genus.”

Structural genes preceded by leader genes at a distance of 10–11 bp contain a variety of domains identified using the Pfam database. However, there is no reason to identify the type of proteins encoded by such structural genes or to link these pairs to a regulation depending on the concentration of a specific amino acid. However, an adjacent leader gene can be involved in the regulation of gene expression. Specifically, ribosome stalling at regulatory codons decreases the rate of ribosome reinitiation.

For example, mRNAs encoding proteins with the PF00271 (Helicase_C) and PF00270 (DEAD) domains in *Corynebacterium diphtheriae* contain the palindromic sequence GCCUUgAAGGC overlapping the stop codon of the leader gene by one nucleotide as well as the whole region upstream of the start codon of the structural gene (positions −11 to −1 relative to the start codon). This RNA region forms a single duplex with the free energy of −4.6 kcal/mol. The RNAs encoding proteins with the same domains in *C. glutamicum* have the Shine-Dalgarno sequence overlapping the hairpin CCgACUAgaguUAGUgGG with the free energy of −6.3 kcal/mol. The RNAs encoding proteins with the same domains in *Bifidobacterium animalis* and *Streptomyces griseus* have the start codon overlapped by the RNA helix containing a G-U pair with the free energy of −1.4 kcal/mol. The hairpin has a long loop as well as a long distance of 17 nt between the stop codon of the leader gene and the start codon of the structural gene. These secondary structures are available in the Additional file 3, part 1.

In general terms, a large number of leader genes without a pronounced abundance of regulatory codons for rare amino acids assumes that most of them are not involved in regulatory mechanisms depending on the concentration of amino acids or aminoacyl-tRNA synthetases.

Some taxonomic groups include relatively few sequenced genomes or can belong to related strains; in this case, the corresponding diagram shows the noise as a large number of low bars; such diagrams are also included in the Additional file 2, part 2. Chlamydiae, Chlorobia, Chloroflexi, Fusobacteria, and Thermotogae exemplify such groups. In these cases, the presence or absence of the peak in a diagram is insignificant. The cases of interest include no or few peaks, which is actually observed in groups with many sequenced genomes representing different genera.

Additional file 3, part 2 shows diagrams plotting frequencies of leader-structural gene pairs for different stop codons of the leader gene in Spirochaetales, Acidobacteria, the Deinococcus–Thermus group, and Planctomycetes. The considered peak was most pronounced for UAG and least for UGA.

### Discussion

Our data on Actinobacteria and Proteobacteria are in good agreement with the high incidence of the attenuation mechanism including its classical variant in them [7]. Taxonomic groups Cyanobacteria, Tenericutes, and Firmicutes, which had no peak or not very pronounced peak, represent early diverged bacterial branches [15, 16]. In addition, there is no reliable data on the presence of attenuation mechanism in cyanobacteria and tenericutes. This also agrees with our hypothesis specified below.

We propose that the leader genes in such proximity to structural genes make up a common operon with them to increase the rate of translation initiation of the structural gene product. In this case, the ribosome can start translation directly or reinitiate translation after translating the leader peptide. Thus, the leader region functions as an “antenna” on a polycistronic mRNA to increase the rate of translation initiations for the structural gene product.

The attenuation mechanism of expression control depending on the concentration of a certain amino acid usually requires a relatively long distance between the leader and structural genes so that the transcription terminator forms outside of mRNA region covered by the ribosome. The ribosome covers up to 12 nt downstream of the stop codon, and the terminator does not overlap the ribosome-binding site of the structural gene; hence, the distance between them should be at least 40 bp. The exact value of this distance is insignificant and is not subject to stabilizing selection. This distance estimate is also confirmed by modeling the attenuation mechanism [2, 6, 7] and by experiments with RNA duplex disrupted by mutation [3].

Different frequencies for different stop codons of leader genes can reflect the involvement of stop codons in ribosome reinitiation. In particular, the rate of ribosome release after translation can differ for stop codons since different stop codons interact with different release factors.

Small quantities of leader genes at a distance of 6–9 bp can be attributed to the overlapping of this region by the purine-rich Shine-Dalgarno sequence, which interferes with the presence of any pyrimidine-containing stop codon here. This effect is valid for all stop codons.

The presence of a typical Shine-Dalgarno sequence in Actinobacteria is confirmed by the sequence logo of the 30-nt 5'-leader regions of all structural genes in Actinobacteria (Additional file 3, part 3).

The masters’ thesis by M. Shchelkunov suggests that the mean intergenic distance decreases with the environmental temperature optimal for bacterial growth. Unfortunately, the described peak is nearly the same in some *Streptomyces* living in the soil as well as symbionts and agents of infectious diseases in homeotherms. Accordingly, the effect difference observed between taxa can hardly be attributed to the temperature.

### Availability of data

Genomic data were extracted from GenBank. Due to its large volume, the tables listing the leader-structural gene pairs for all annotated bacterial genes available in GenBank can be accessed at [11].

## Reviewers' comments

*We took the liberty to number the review paragraphs to place our answers to comments of the following numbers.*

### Reviewer's report 1: Piotr Zielenkiewicz, Institute of Biochemistry and Biophysics PAS, Poland

## Reviewer comments

1. In their manuscript entitled “Ribosome Reinitiation at Leader Peptides Increases Translation of Bacterial Proteins” Korolev and coworkers present the hypothesis that so-called leader genes (typically small open reading frames that presumably can regulate translation of a downstream gene through a translation-based modification of secondary structure of mRNA) increase the initiation rate of translation of downstream genes in several bacterial genera. I have several doubts about this work, as it is largely echoing authors’ previous work and doesn’t take into account other available data.

Response: *We have presented reports at two local conferences, which only marginally concerned the problem of “the short distance between the leader and first structural genes”; the theses were only published in* [17, 18]. *We have no other publications on this problem*.

*We have a long-term interest in the attenuation mechanism, which led us to the study of the short distance problem. We were amazed that, in the case of the short distance, all variants of the attenuation mechanism seem impossible considering that the modification of mRNA secondary structure looks forbidden.*

*We are unaware of “other available data” on this problem (see also the response 2 below).*

2. Major issues:

No attempt to confront the observed distance between leader genes and downstream genes and the observed distance in known operons has been made. In Escherichia coli the distance between two consecutive genes peaks at around 10 nt.

While it might support authors’ hypothesis (and there is a glimpse of such discussion in the text), there is no discussion of other mechanisms putatively involved.

Response: *We assume no direct relationship between the distance from the leader gene to the first structural one, on the one hand, and the distance between neighboring structural genes, on the other hand. The latter distance problem becomes even more complicated if it is analyzed specifically for operons. We are grateful to the reviewer for setting out the former distance problem in the context of the distance between neighboring genes in general in bacterial operons. We have bioinformatics data relevant to the latter distance problem. Several examples of this kind composed the new Additional file**4**: the distances between neighboring structural genes for individual model species and taxonomic groups. Specifically, there is a pronounced peak for this distance of 10–11 bp, a minor peak for the distance of 13–16 bp, and no peak for positive distances in Escherichia coli K-12, Bacillus subtilis 168 and Synechocystis sp. PCC 6803 (NC_000911). This pattern is similar to that revealed for leader genes. In all cases of data averaged for many species, there is a peak between neighboring structural genes for the distance of 2–4 bp, which is not observed for leader genes. The distances in the range of 6–9 bp are rare. The frequency of pairs of neighboring structural genes at the distance of 10–13 bp has a weak local maximum of considerable width. The local maximum falls on the distance of 10–11 bp in Actinobacteria and Proteobacteria; 10 bp, in Spirochaetales and Cyanobacteria; and 13 bp, in Firmicutes (which is particularly wide in this case). In Firmicutes, the position of the maximum roughly corresponds to that for leader genes (with no account of cyanobacteria). The diagram for Proteobacteria is distant from that for E. coli. Thus, the peak can be pronounced in some cases or missing in other ones. We plan to consider this problem in a separate report not focused on the attenuation mechanism*.

*Unfortunately, we failed to find other mechanisms that could underlie the described effect, although they are of clear interest (see also the response to the second reviewer below).*

3. If leader genes indeed increase re-initiation of translation of downstream genes, such an effect should be visible in ribosome profiling studies. No analysis of available data has been made (there are three bacterial genomes available at GWIPS database if authors don’t want to make their own re-analysis of ribosome profiling experiments).

Response: *Ref.* [19] *(extracted from the GWIPS database) describes ribosome profiling as “the ribosome density along all mRNA transcripts present in the cell to be quantified” and presents ribosome profiling for two bacterial species (E. coli and B. subtilis). The data on the operon translation in the wild type and in a strain with a mutation in a leader gene can be used to verify our bioinformatics predictions, in particular, using ribosome profiling. Extracting the data for the mutant from ribosome profiling experiments requires further (and possibly non-bioinformatics) study*.

4. I didn’t find such analysis in previous works of the authors either.

Response: *See the response 1*.

5. There is no discussion about results varying from branch to branch of bacterial taxonomy. No attempt to explain the differences in the context of phenotypic traits of distinct species nor in the context of individual genes being regulated.

Response: *The discussion of the observed effect for early diverged or remote species can be found in the Discussion Section. A discussion “in the context of phenotypic traits of distinct species” (i.e., in a general biological context) presents the usual difficulties. Clarification concerning the environmental temperature for bacteria was added to the Discussion Section (paragraph 7) to address the comment by the reviewer 2. The effect “in the context of individual genes being regulated” is discussed in paragraph 7 of the Results Section and in the Additional file**3**(part 1). We presume that the effect in most cases is mediated by the reinitiation, which can have a regulatory importance per se, but is unrelated to the attenuation mechanism. This point concerning the role of leader genes is further supported in the text*.

6. Overall, the manuscript seems to be nothing more than an early draft and needs a substantial amount of work. The context is lacking (background is limited to citing mainly authors’ own papers), there is no attempt to confirm the obtained results by using external data and finally, no alternative hypotheses are considered.

Response: *We believe that the reported observation is new and adequately supported at the bioinformatics level. It is negatively related to the attenuation mechanism, which is discussed in the Introduction of this nonetheless short report. We are unaware of the “external data” and cannot propose “alternative hypotheses” relevant to the problem; although both are of great interest*.

### Reviewer's report 2: István Simon, Institute of Enzymology, Hungary

## Reviewer comments

This is a nice survey of the lead genes and their distance from the protein coding (structure) genes. The authors provide an interesting discussion on the observations. However, I should like to see a few additional discussions: They observed that for most bacteria the distance distribution exhibit the highest value around ten base pairs. Ten base pairs is the size of one turn in the double helix, so after ten base pairs the orientation of the last base pair is the same as the first one. Can it be a reason of their finding? For Thermotogae bacterium, an opposite distribution was find. At the distance of ten base pairs a minimum was observed. Thermotogae bacteria live at high temperature, where most DNA has limited structural stability. Can it be the reason of the opposite distance distribution? I recommend the publication of this manuscript after minor revision.

Response: *Considering that the distance between the leader and the first structural gene is similar to the length of one DNA turn, it is tempting to propose a mechanism involving gene transcription; however, we failed to lay it down. The response to the second comment was added to the Discussion Section (paragraph 7)*.

## Additional files

Additional file 1: Lists of used genomes and taxonomic groups of bacteria. (XLSX 120 kb) Additional file 2: Part 1. Plots of the mean length (calculated on the base of our data) of leader genes as a function of their distance from the structural gene for the following taxonomic groups. Figure S1.1. Actinobacteria. Figure S1.2. Bacteroides. Figure S1.3. Cyanobacteria; Part 2. Diagrams plotting the number of leader genes as a function of their distance from the structural gene for the following taxonomic groups. Figure S2.1. All Actinobacteria; Figure S2.2. All Actinobacteria (one genome per genus); Figure S2.3. Corynebacterineae; Figure S2.4. *Corynebacterium*; Figure S2.5. Bifidobacteriales; Figure S2.6. *Bifidobacterium*; Figure S2.7. *Streptomyces*; Figure S2.8. *Mycobacterium*; Figure S2.9. Other Actinobacteria; Figure S2.10. All Proteobacteria; Figure S2.11. All Proteobacteria (one genome per genus); Figure S2.12. Alpha-proteobacteria (one genome per genus); Figure S2.13. Beta-proteobacteria (one genome per genus); Figure S2.14. Delta-proteobacteria (one genome per genus); Figure S2.15. Epsilon-proteobacteria (one genome per genus); Figure S2.16. Gamma-proteobacteria (one genome per genus); Figure S2.17. All Firmicutes; Figure S2.18. All Firmicutes (one genome per genus); Figure S2.19. All Bacteroides; Figure S2.20. All Bacteroides (one genome per genus); Figure S2.21. All Spirochaetales; Figure S2.22. Acidobacteria; Figure S2.23. Aquificaceae; Figure S2.24. Chlamydiae; Figure S2.25. Chlorobia; Figure S2.26. Chloroflexi; Figure S2.27. Cyanobacteria; Figure S2.28. Deinococcus–Thermus group; Figure S2.29. Fusobacteria; Figure S2.30. Planctomycetes; Figure S2.31. Tenericutes; Figure S2.32. Thermotogae. (PDF 288 kb) Additional file 3: Part 1. RNA secondary structures of 5'-untranslated regions of proteins with the PF00270 and PF00271 domains in *Corynebacterium diphtheria*, *C. glutamicum*, and *Bifidobacterium animalis*. Figure S1.1. RNA duplex in the region from the stop codon of the leader gene to the start codon of the structural gene encoding helicase in *C. diphtheria*; Figure S1.2. RNA hairpin overlapping the Shine-Dalgarno sequence in the helicase in *C. glutamicum*; Figure S1.3. RNA hairpin overlapping two nucleotides of the helicase start codon in *B. animalis* and *Streptomyces griseus*. Part 2. Frequency of the leader-structural gene pairs as a function of the leader gene stop codon in Spirochaetales, Acidobacteria, Deinococcus-Thermus group, and Planctomycetes. Figure S2.1. Acidobacteria; Figure S2.2. Deinococcus–Thermus group; Figure S2.3. Planctomycetes; Figure S2.4. Spirochaetales; Figure S2.5. Actinobacteria. Part 3. Sequence logo of the 30-nt 5'-leader regions of all structural genes in Actinobacteria. (PDF 365 kb) Additional file 4: Part 1. Frequency plots for the distance between two adjacent structural genes in *Escherichia coli* K-12, *Bacillus subtilis* 168 and *Synechocystis* sp. PCC 6803. Part 2. Frequency plots for the distance between two adjacent structural genes in Actinobacteria, Cyanobacteria, Firmicutes, Proteobacteria, and Spirochaetales. (PDF 126 kb)

## Abbreviations

bp: base pairs
DNA: deoxyribonucleic acid
NCBI: National Center for Biotechnology Information
mRNA: messenger ribonucleic acid
nt: nucleotides
RNA: ribonucleic acid
tRNA: transfer ribonucleic acid

## References

[1] Seliverstov AV, Putzer H, Gelfand MS, Lyubetsky VA (2005). Comparative analysis of RNA regulatory elements of amino acid metabolism genes in Actinobacteria. BMC Microbiol..

[2] Lyubetsky VA, Korolev SA, Seliverstov AV, Zverkov OA, Rubanov LI (2014). Gene expression regulation of the PF00480 or PF14340 domain proteins suggests their involvement in sulfur metabolism. Comput Biol Chem..

[3] Das A, Crawford IP, Yanofsky C (1982). Regulation of tryptophan operon expression by attenuation in cell-free extracts of *Escherichia coli*. J Biol Chem.

[4] Heery DM, Dunican LK (1993). Cloning of the *trp* gene cluster from a tryptophan hyperproducing strain of *Corynebacterium glutamicum*: Identification of a mutation in the *trp* leader sequence. Appl Environ Microbiol.

[5] Lin C, Paradkar AS, Vining LC (1998). Regulation of an anthranilate synthase gene in *Streptomyces venezuelae* by a *trp* attenuator. Microbiology.

[6] Lyubetsky VA, Pirogov SA, Rubanov LI, Seliverstov AV (2007). Modeling classic attenuation regulation of gene expression in bacteria. J Bioinform Comput Biol.

[7] Lopatovskaya KV, Seliverstov AV, Lyubetsky VA (2010). Attenuation Regulation of the Amino Acid and Aminoacyl-tRNA Biosynthesis Operons in Bacteria: A Comparative Genomic Analysis. Mol Bio.

[8] Glotova I, Rubanov LI, Seliverstov AV, Lyubetsky VA. Pseudoknots and RNA triplexes in the classical attenuation regulation model [in Russian]. Proceedings of the 30th conference Information Technologies and Systems (ITaS’07), Zvenigorod, September 18–21, 2007, p. 222–227.

[9] Luo H, Gilinger G, Mukherjee D, Bellofatto V (1999). Transcription Initiation at the TATA-less Spliced Leader RNA Gene Promoter Requires at Least Two DNA-binding Proteins and a Tripartite Architecture That Includes an Initiator Element. J Biol Chem.

[10] Schuwirth BS, Borovinskaya MA, Hau CW, Zhang W, Vila-Sanjurjo A, Holton JM, Doudna Cate JH (2005). Structures of the Bacterial Ribosome at 3.5 Å Resolution. Science..

[11] Predicted leader–structural gene pairs for all annotated bacterial genes available in the NCBI database. http://lab6.iitp.ru/-/leader_genes. Accessed 10 Feb 2016.

[12] Finn RD, Bateman A, Clements J, Coggill P, Eberhardt RY, Eddy SR, Heger A, Hetherington K, Holm L, Mistry J, Sonnhammer ELL, Tate J, Punta M (2014). The Pfam protein families database. Nucleic Acids Res..

[13] Crooks GE, Hon G, Chandonia JM, Brenner SE (2004). WebLogo: A sequence logo generator. Genome Res..

[14] Reuter JS, Mathews DH (2010). RNAstructure: software for RNA secondary structure prediction and analysis. BMC Bioinform..

[15] Lyubetsky VA, Rubanov LI, Rusin LY, Gorbunov KY (2012). Cubic time algorithms of amalgamating gene trees and building evolutionary scenarios. Biol Direct..

[16] Lang JM, Darling AE, Eisen JA (2013). Phylogeny of Bacterial and Archaeal Genomes Using Conserved Genes: Supertrees and Supermatrices. PLoS ONE.

[17] Korolev SA, Seliverstov AV, Zverkov OA, Lyubetsky VA (2015). Tryptophan concentration dependent classical attenuator regulation in Actinobacteria. Mod Inf Tech IT Educ.

[18] Korolev SA, Lyzhin SA, Zverkov OA, Seliverstov AV, Lyubetsky VA. A Search for Genes Encoding Histidine-Containing Leader Peptides in Actinobacteria. Proceedings of the 39th IITP RAS Interdisciplinary Conference & School “Information Technology and Systems 2015”. Moscow: IITP; 2015; 53–60.

[19] Michel AM, Fox G, Kiran AM, De Bo C, O’Connor PBF, Heaphy SM, Mullan JPA, Donohue CA, Higgins DG, Baranov PV (2014). GWIPS-viz: development of a ribo-seq genome browser. Nucleic Acids Res.

