# Supplementary material for: Ribosome reinitiation at leader peptides increases translation of bacterial proteins
Source: Biol Direct. 2016 Apr 16;11:20. doi: 10.1186/s13062-016-0123-8 (PMC4833913; doi:10.1186/s13062-016-0123-8)
Supplement: Additional file 2: — Part 1. Plots of the mean length (calculated on the base of our data) of leader genes as a function of their distance from the structural gene for the following taxonomic groups. Figure S1.1. Actinobacteria. Figure S1.2. Bacteroides. Figure S1.3. Cyanobacteria; Part 2. Diagrams plotting the number of leader genes as a function of their distance from the structural gene for the following taxonomic groups. Figure S2.1. All Actinobacteria; Figure S2.2. All Actinobacteria (one genome per genus); Figure S2.3. Corynebacterineae; Figure S2.4. Corynebacterium; Figure S2.5. Bifidobacteriales; Figure S2.6. Bifidobacterium; Figure S2.7. Streptomyces; Figure S2.8. Mycobacterium; Figure S2.9. Other Actinobacteria; Figure S2.10. All Proteobacteria; Figure S2.11. All Proteobacteria (one genome per genus); Figure S2.12. Alpha-proteobacteria (one genome per genus); Figure S2.13. Beta-proteobacteria (one genome per genus); Figure S2.14. Delta-proteobacteria (one genome per genus); Figure S2.15. Epsilon-proteobacteria (one genome per genus); Figure S2.16. Gamma-proteobacteria (one genome per genus); Figure S2.17. All Firmicutes; Figure S2.18. All Firmicutes (one genome per genus); Figure S2.19. All Bacteroides; Figure S2.20. All Bacteroides (one genome per genus); Figure S2.21. All Spirochaetales; Figure S2.22. Acidobacteria; Figure S2.23. Aquificaceae; Figure S2.24. Chlamydiae; Figure S2.25. Chlorobia; Figure S2.26. Chloroflexi; Figure S2.27. Cyanobacteria; Figure S2.28. Deinococcus–Thermus group; Figure S2.29. Fusobacteria; Figure S2.30. Planctomycetes; Figure S2.31. Tenericutes; Figure S2.32. Thermotogae. (PDF 288 kb) [file 13062_2016_123_MOESM2_ESM.pdf]

Part 1. Plots of the mean length (calculated on the base of our data) of leader genes as a function of their distance from the structural gene for the following taxonomic groups

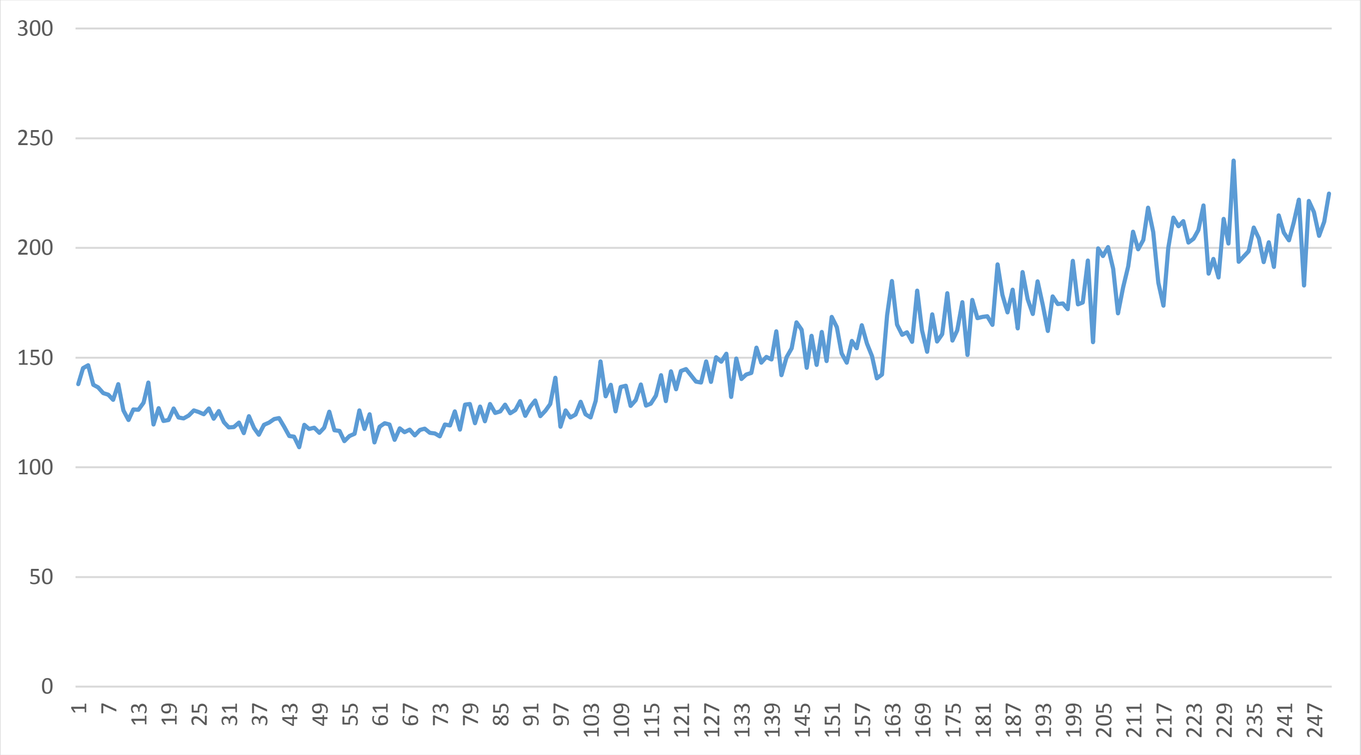

Figure 1.1. Actinobacteria

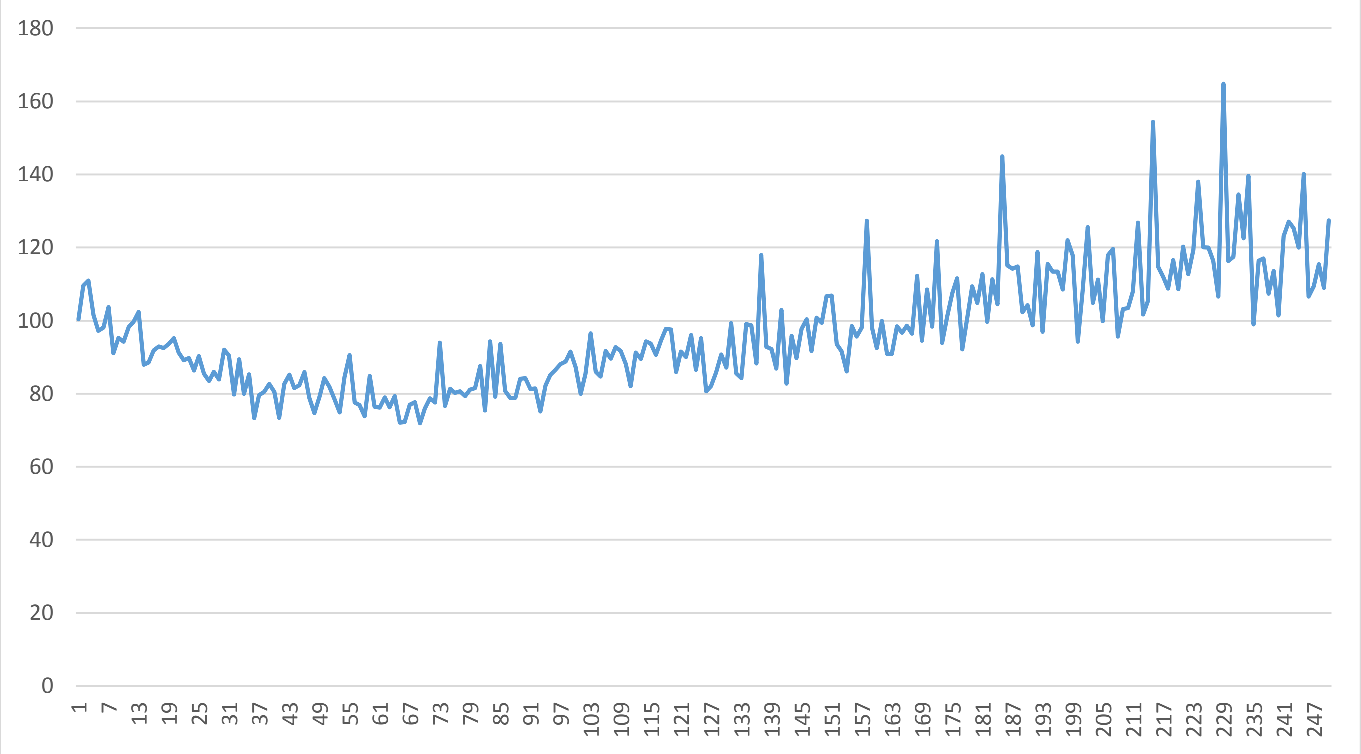

Figure 1.2. Bacteroides

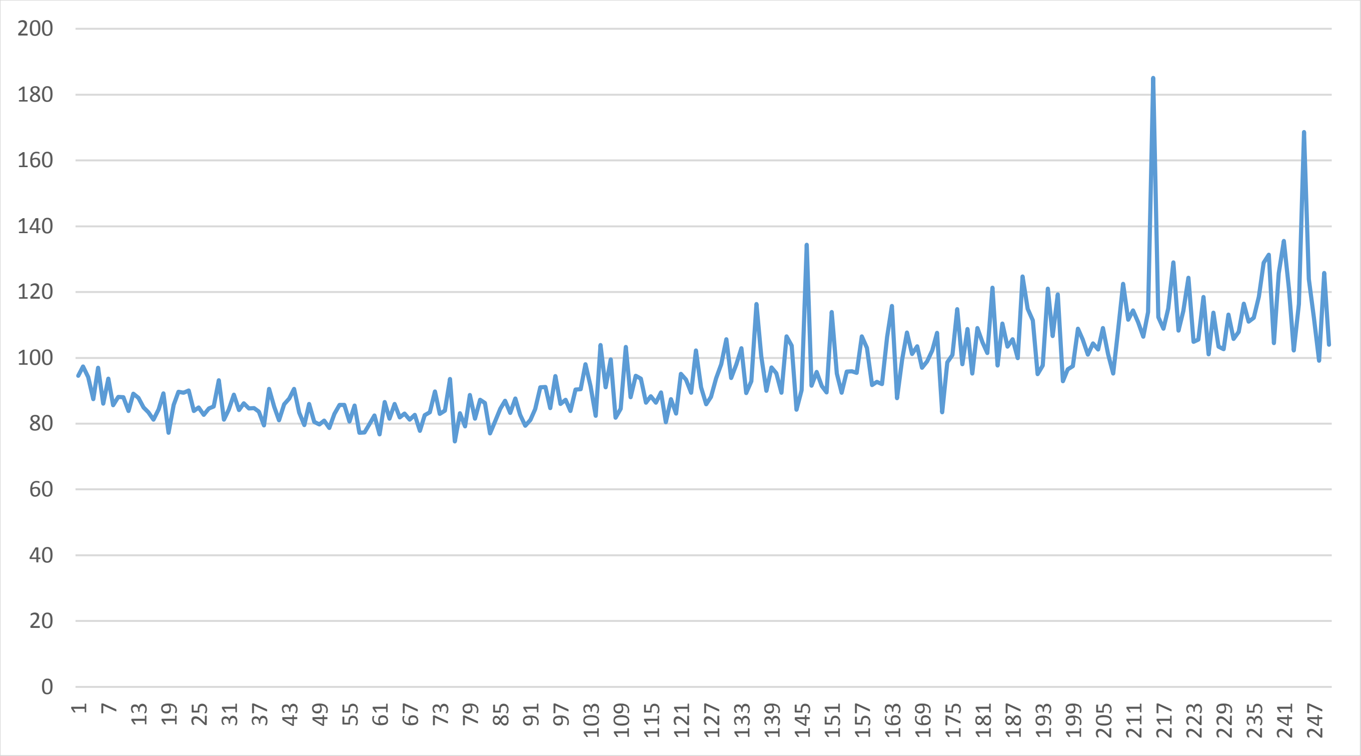

Figure 1.3. Cyanobacteria

Part 2. Diagrams plotting the number of leader genes as a function of their distance from the structural gene for the following taxonomic groups

Actinobacteria

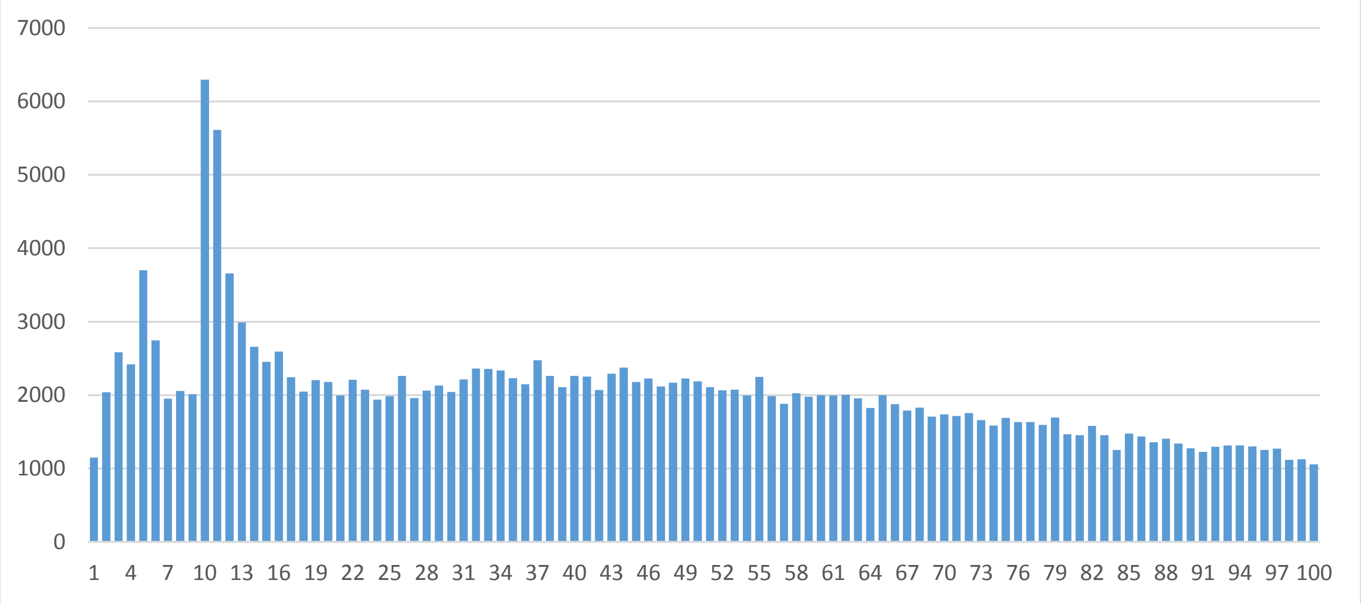

Figure 2.1. All Actinobacteria

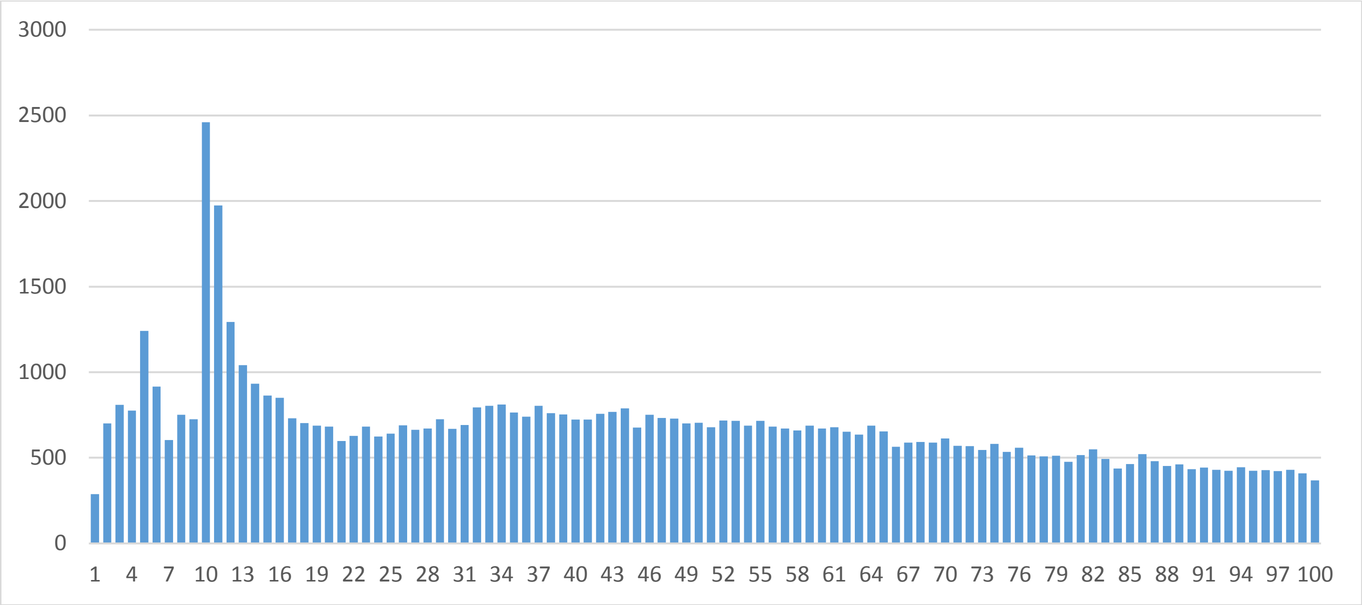

Figure 2.2. All Actinobacteria (one genome per genus)

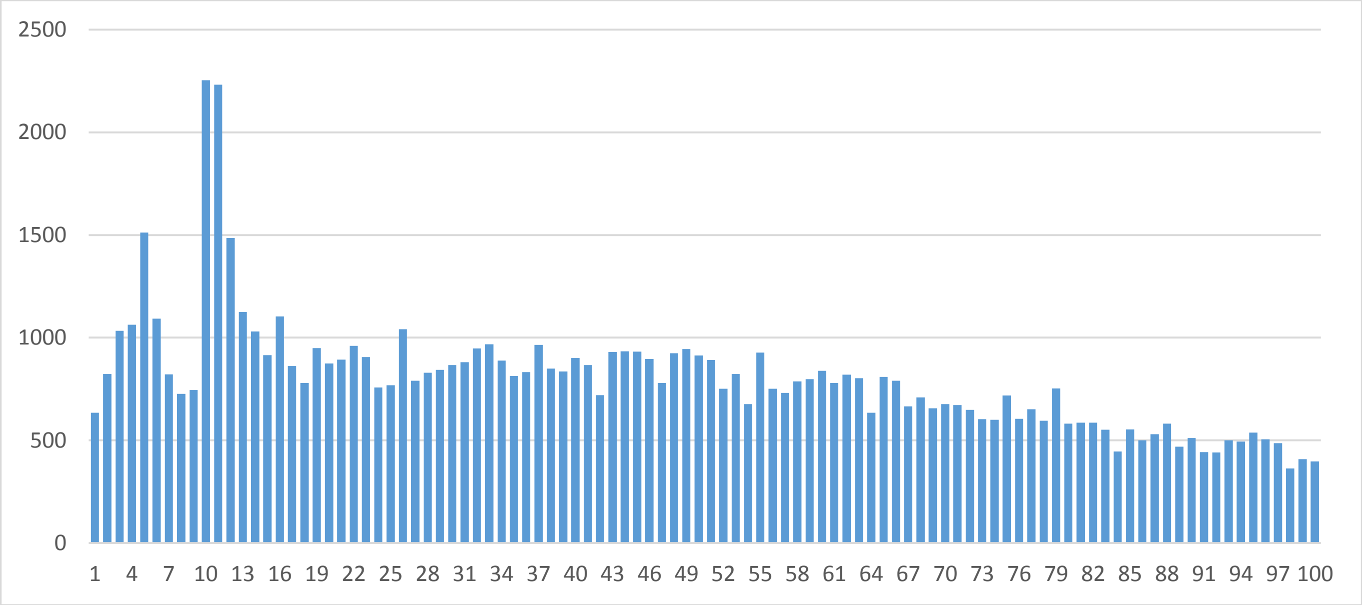

Figure 2.3. Corynebacterineae

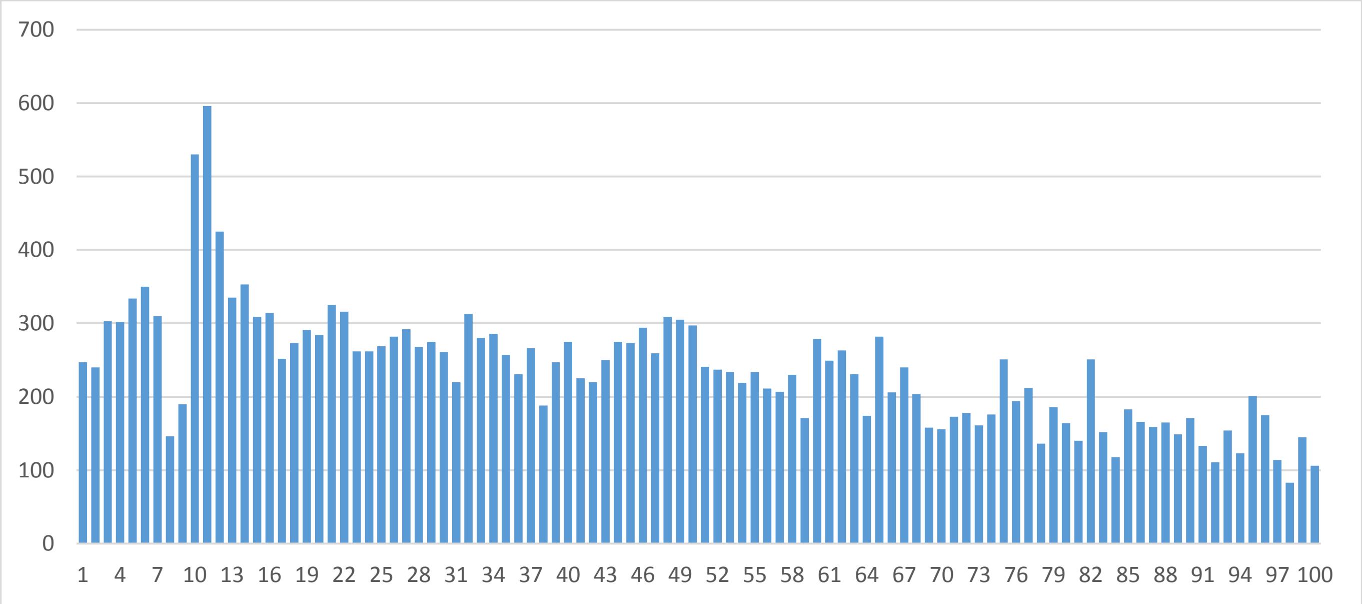

Figure 2.4. *Corynebacterium*

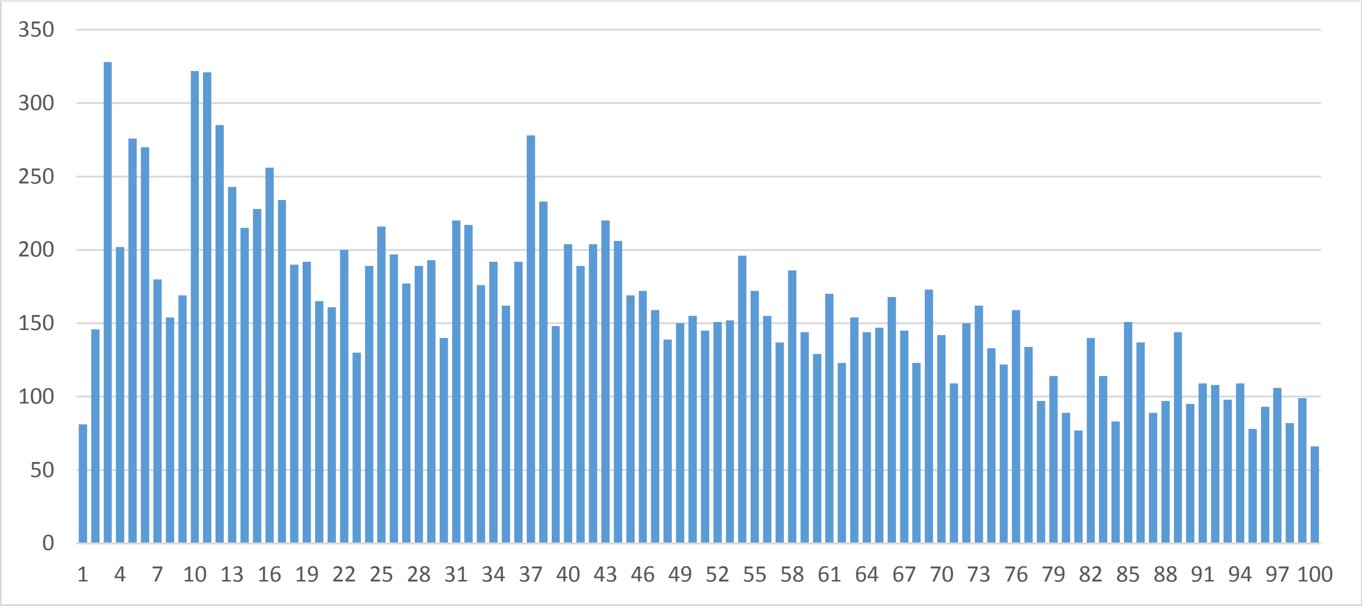

Figure 2.5. Bifidobacteriales

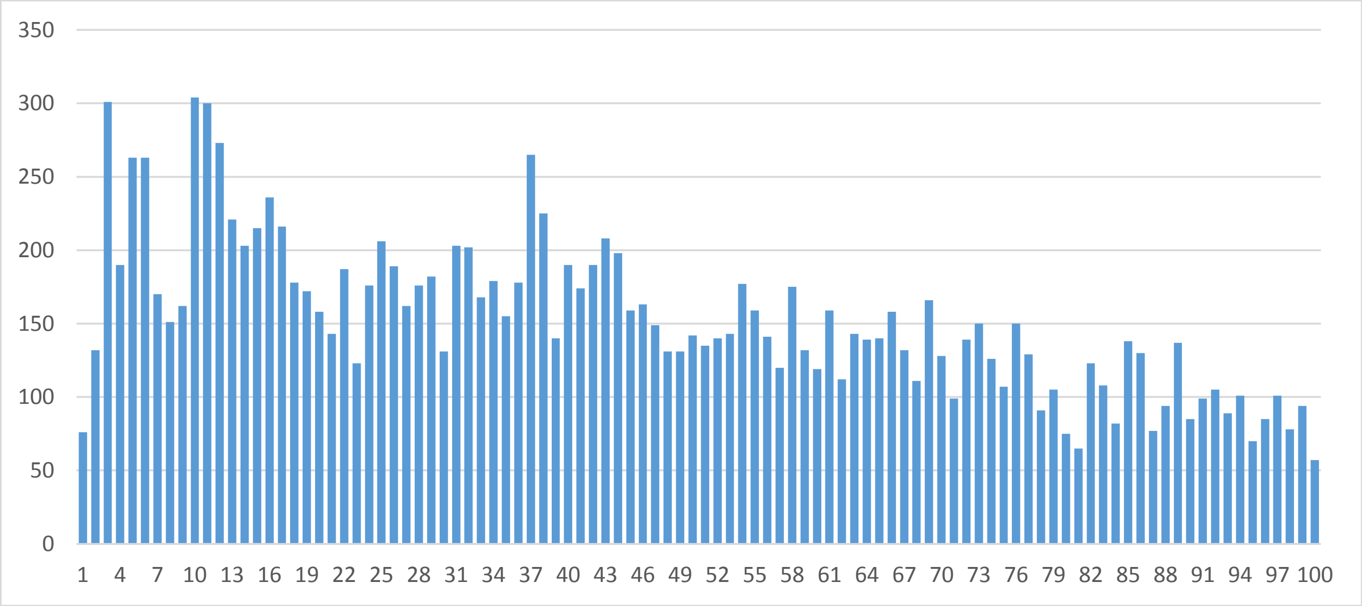

Figure 2.6. *Bifidobacterium*

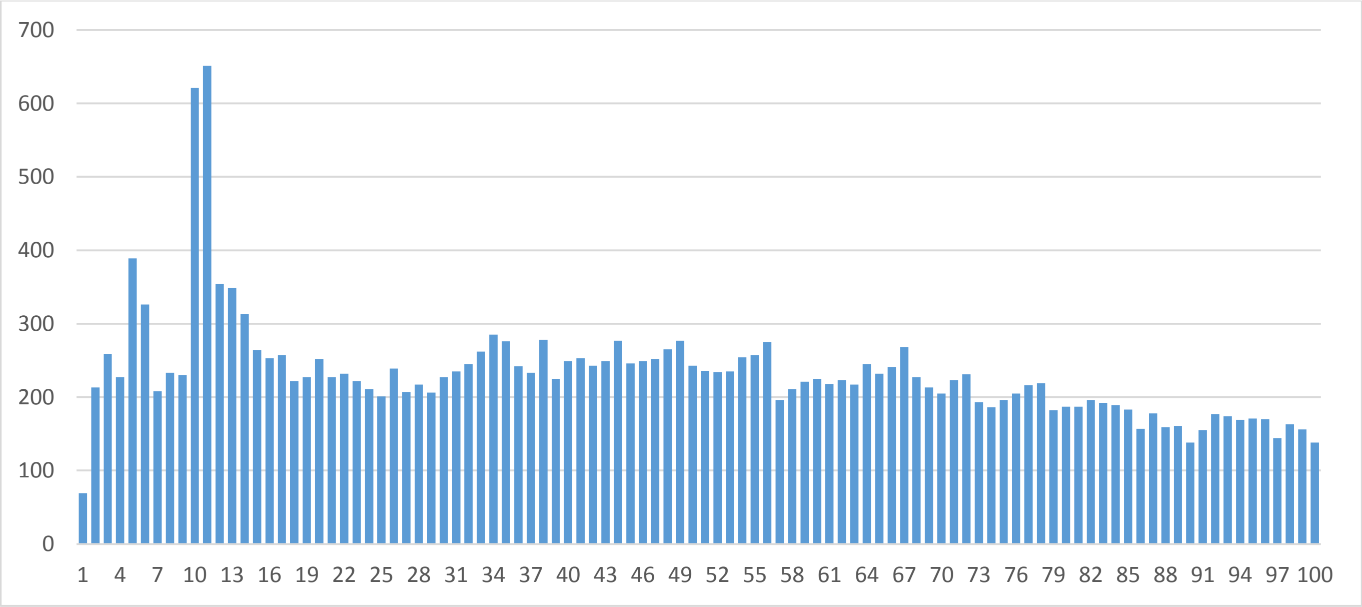

Figure 2.7. *Streptomyces*

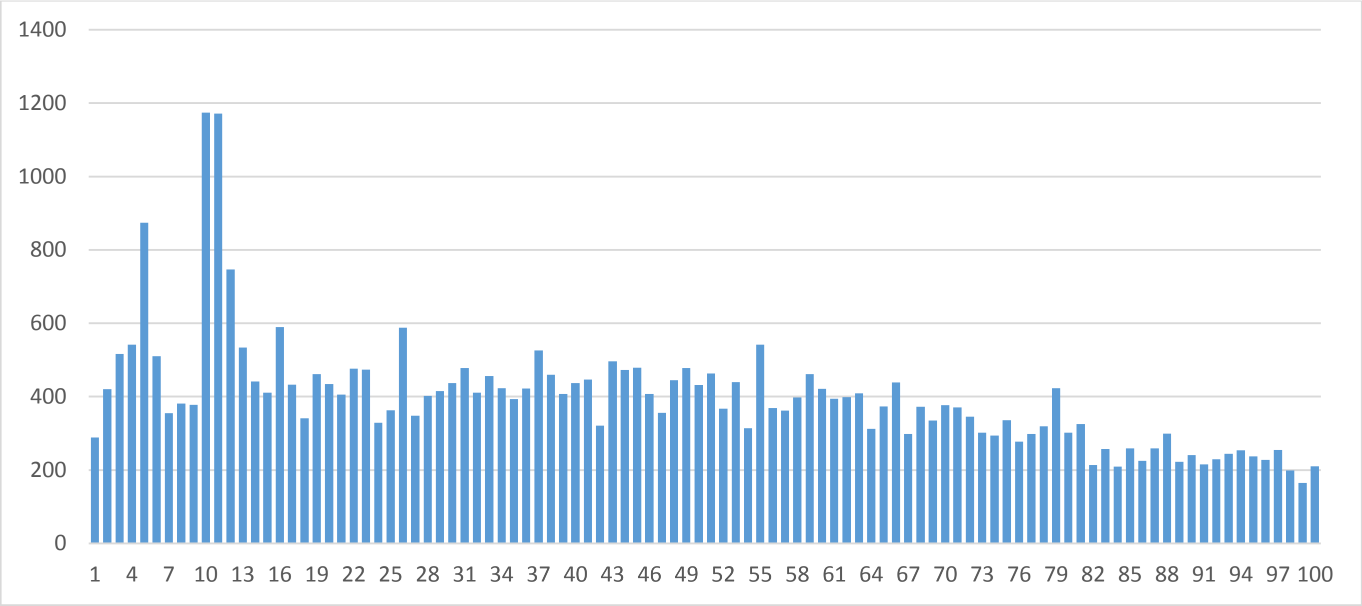

Figure 2.8. *Mycobacterium*

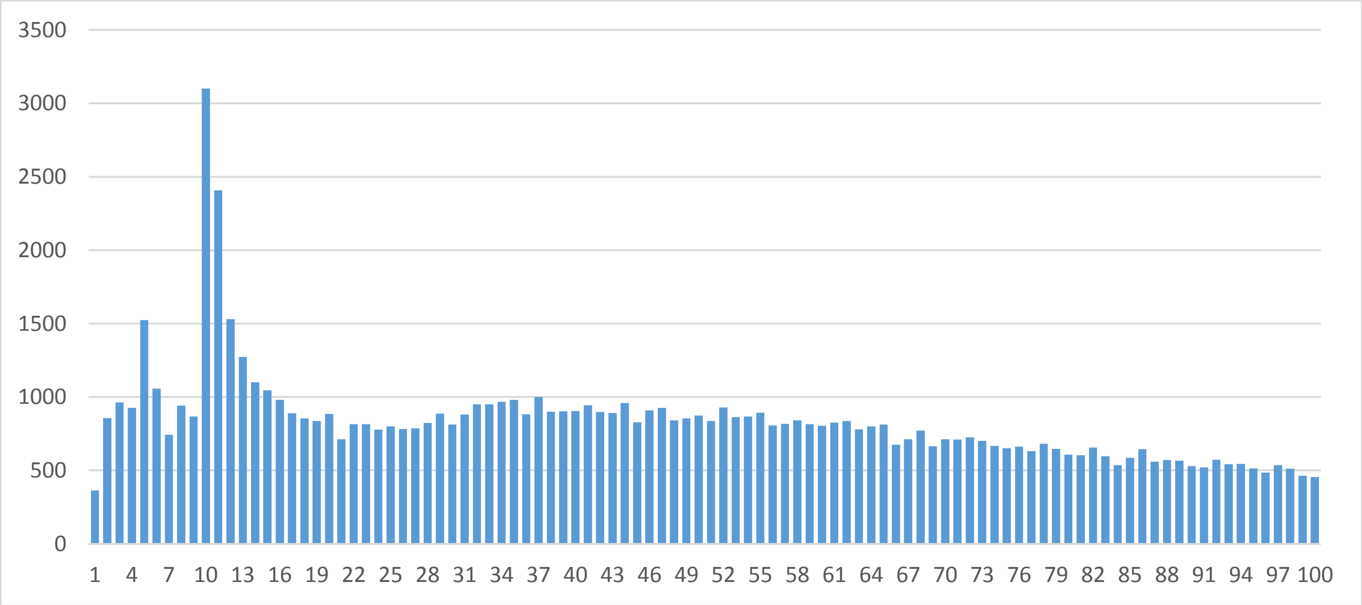

Figure 2.9. Other Actinobacteria

Proteobacteria

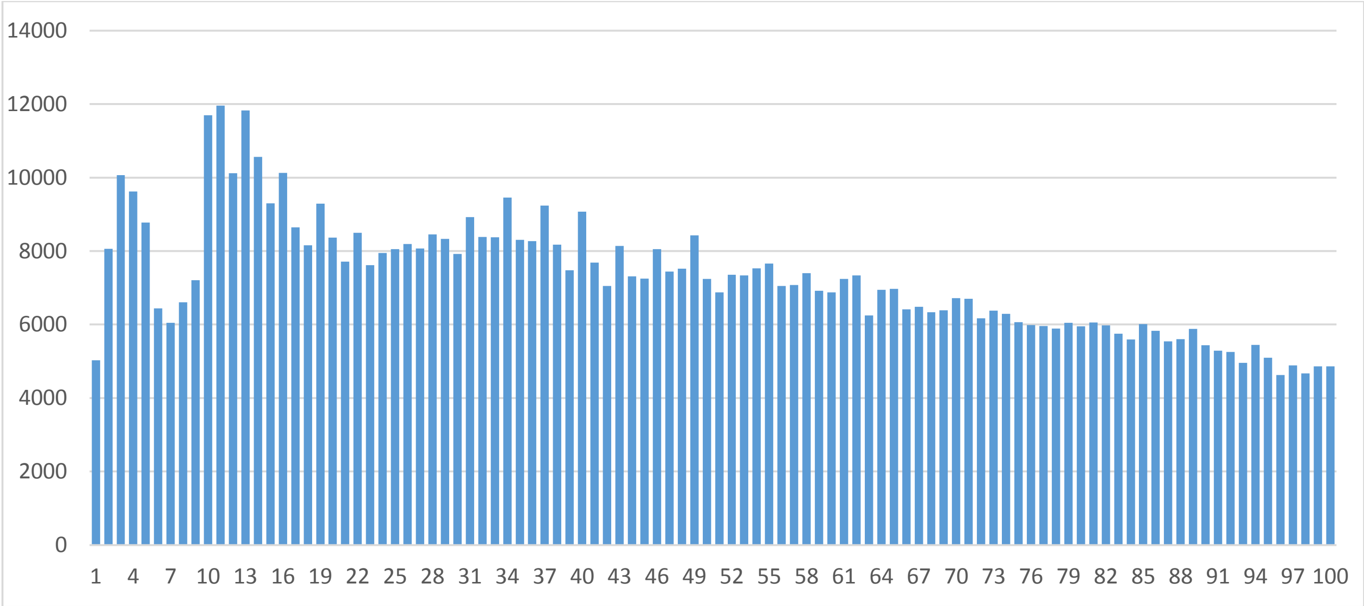

Figure 2.10. All Proteobacteria

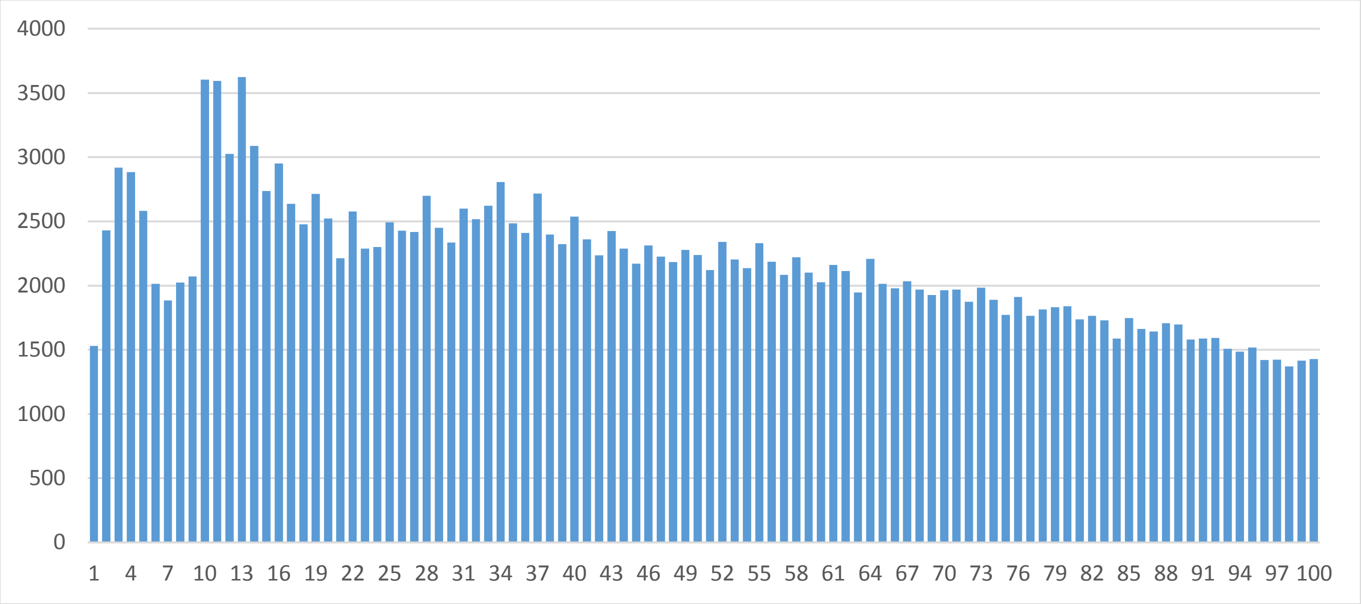

Figure 2.11. All Proteobacteria (one genome per genus)

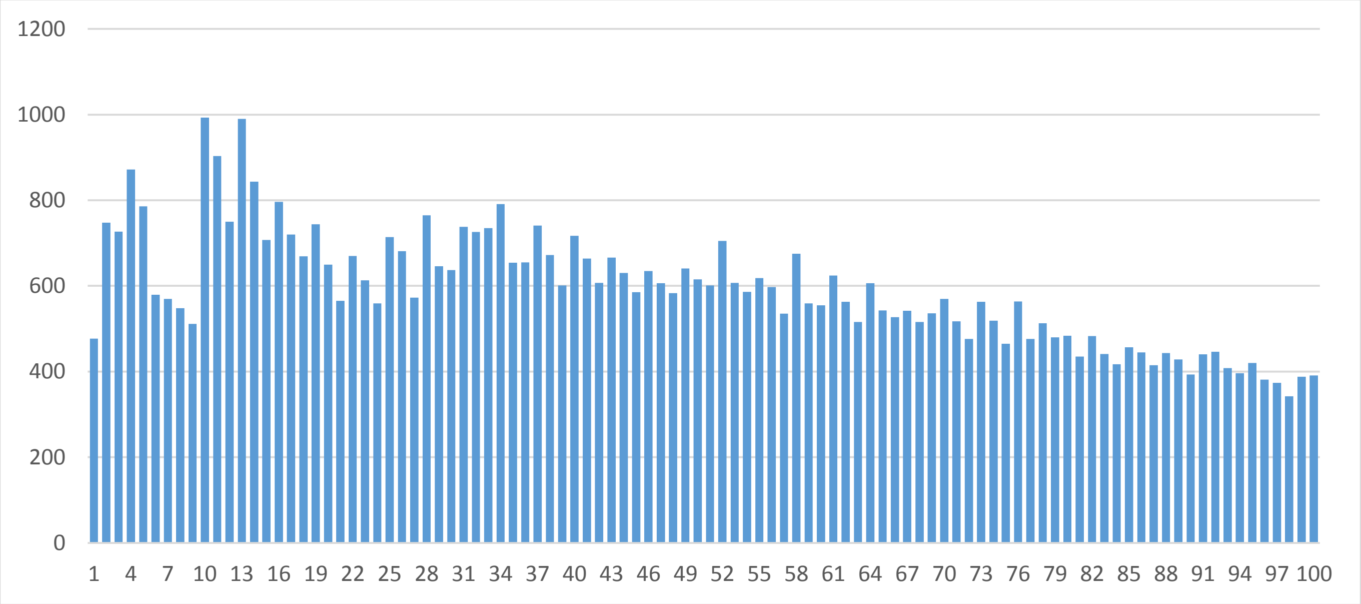

Figure 2.12. Alphaproteobacteria (one genome per genus)

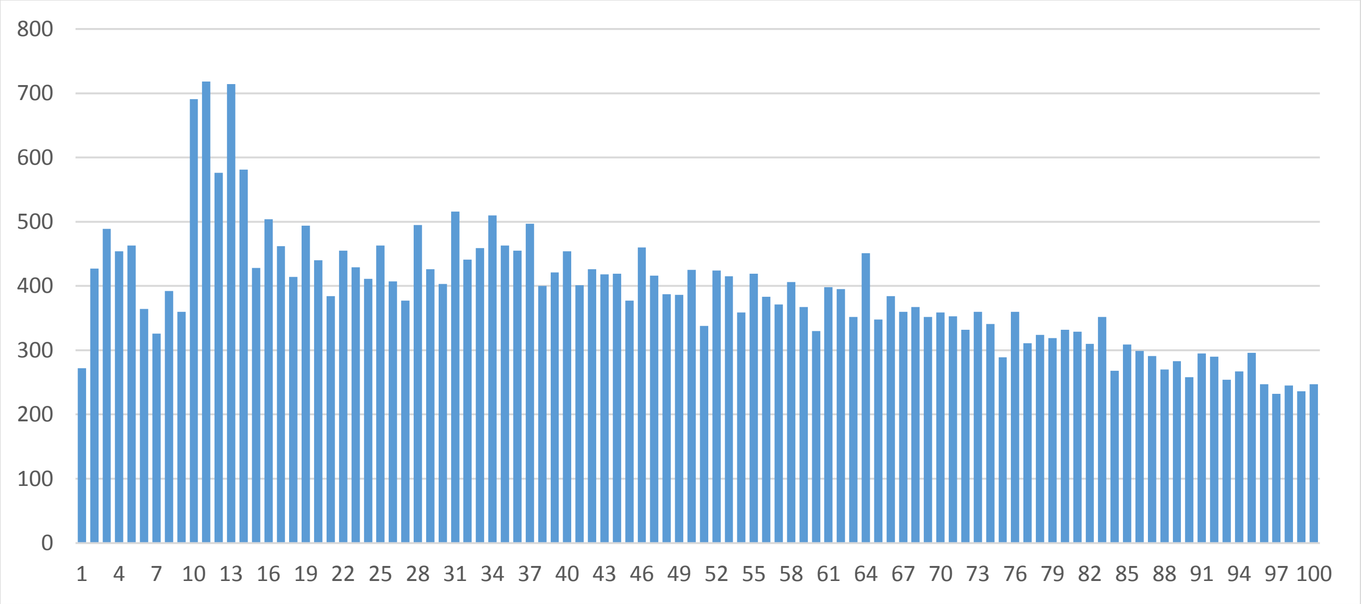

Figure 2.13. Betaproteobacteria (one genome per genus)

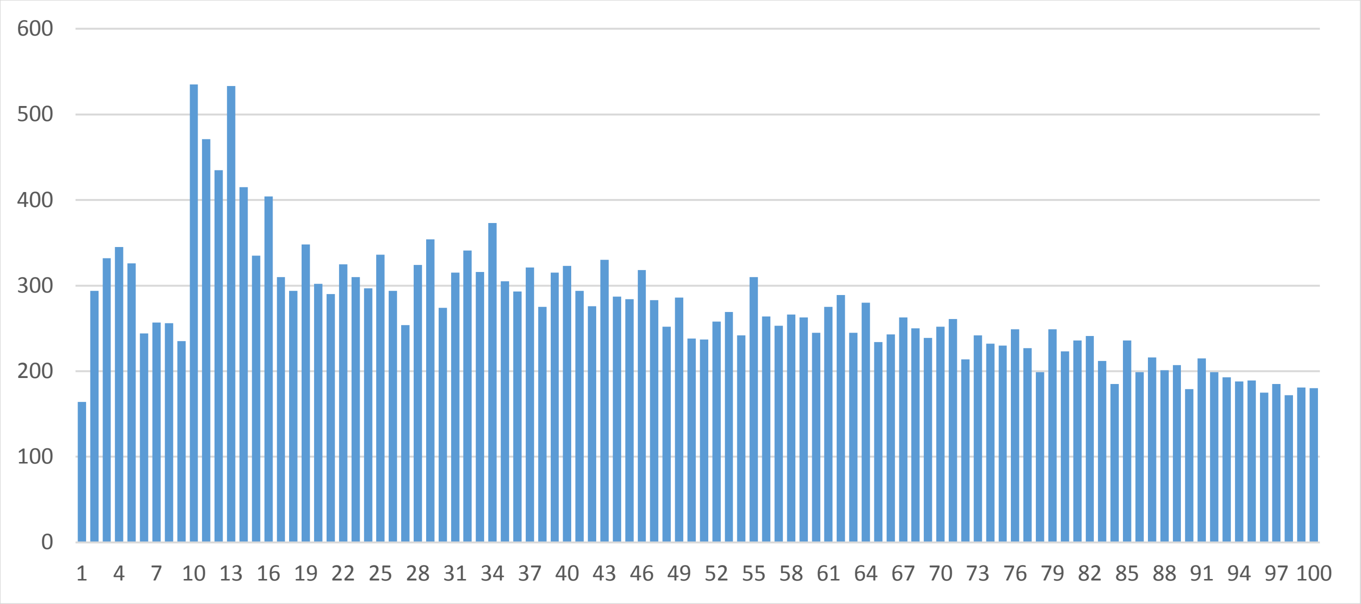

Figure 2.14. Deltaproteobacteria (one genome per genus)

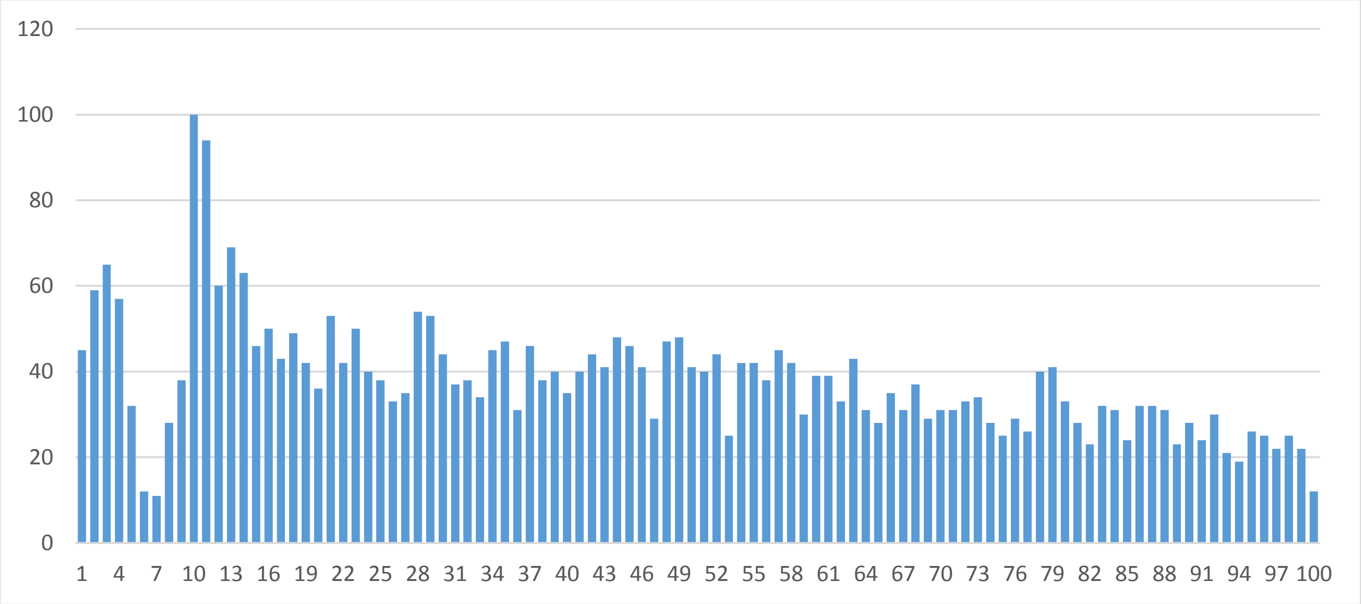

Figure 2.15. Epsilonproteobacteria (one genome per genus)

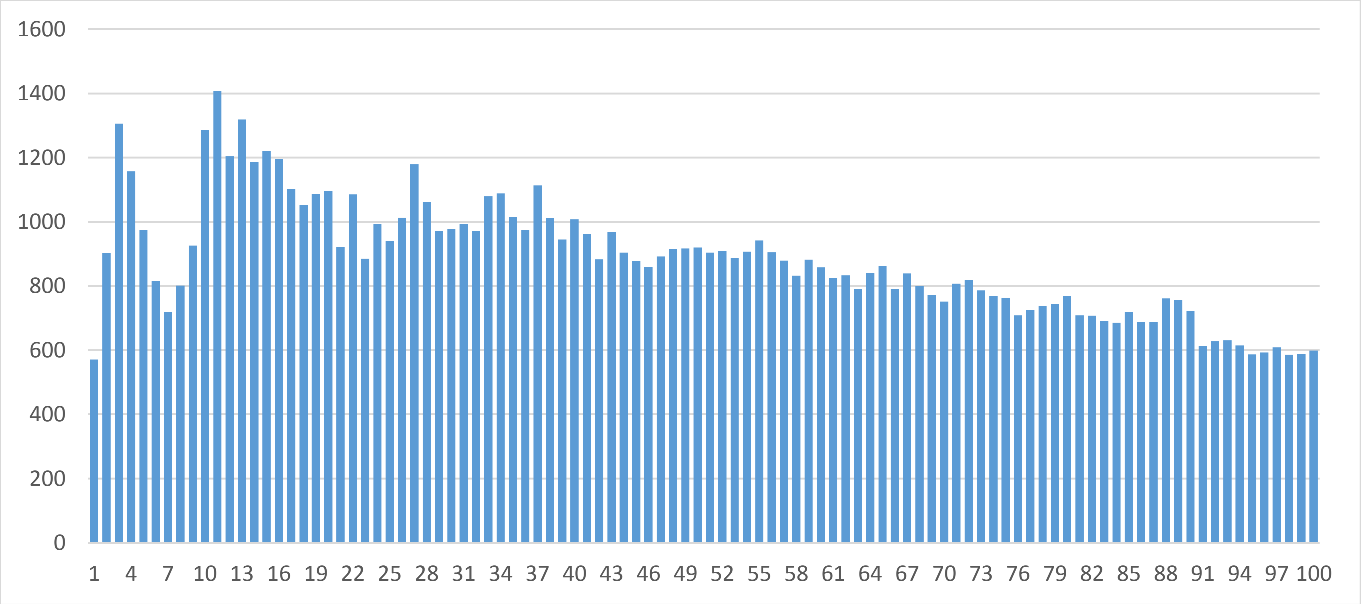

Figure 2.16. Gammaproteobacteria (one genome per genus)

Firmicutes

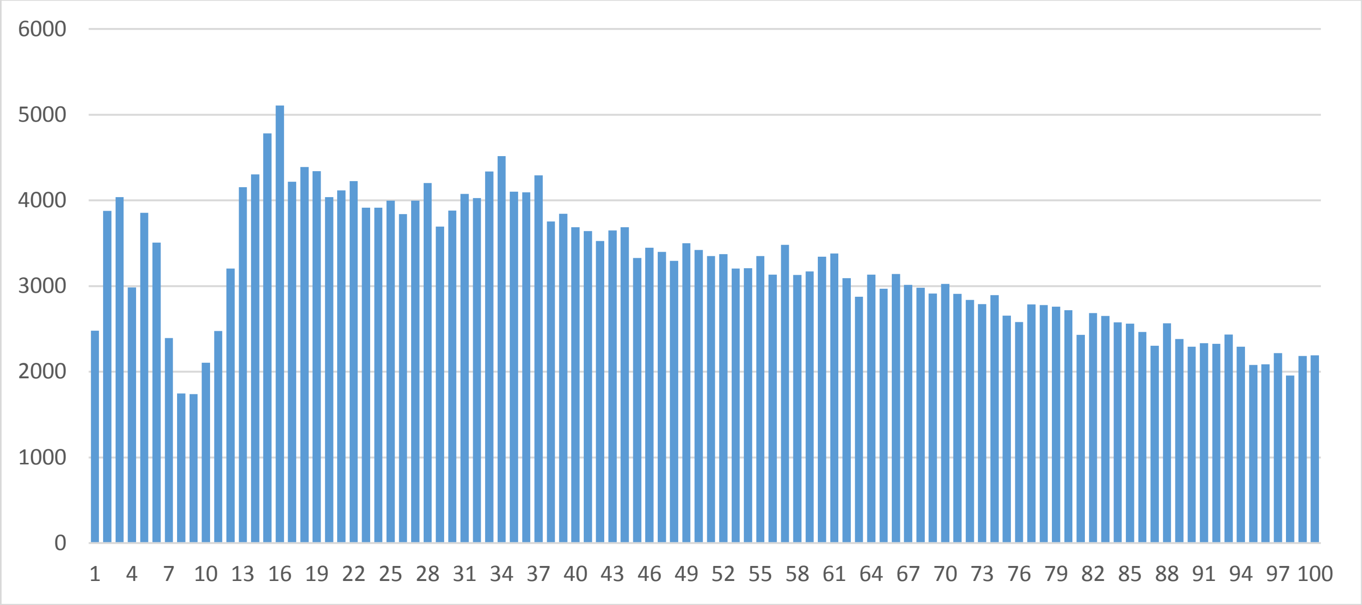

Figure 2.17. All Firmicutes

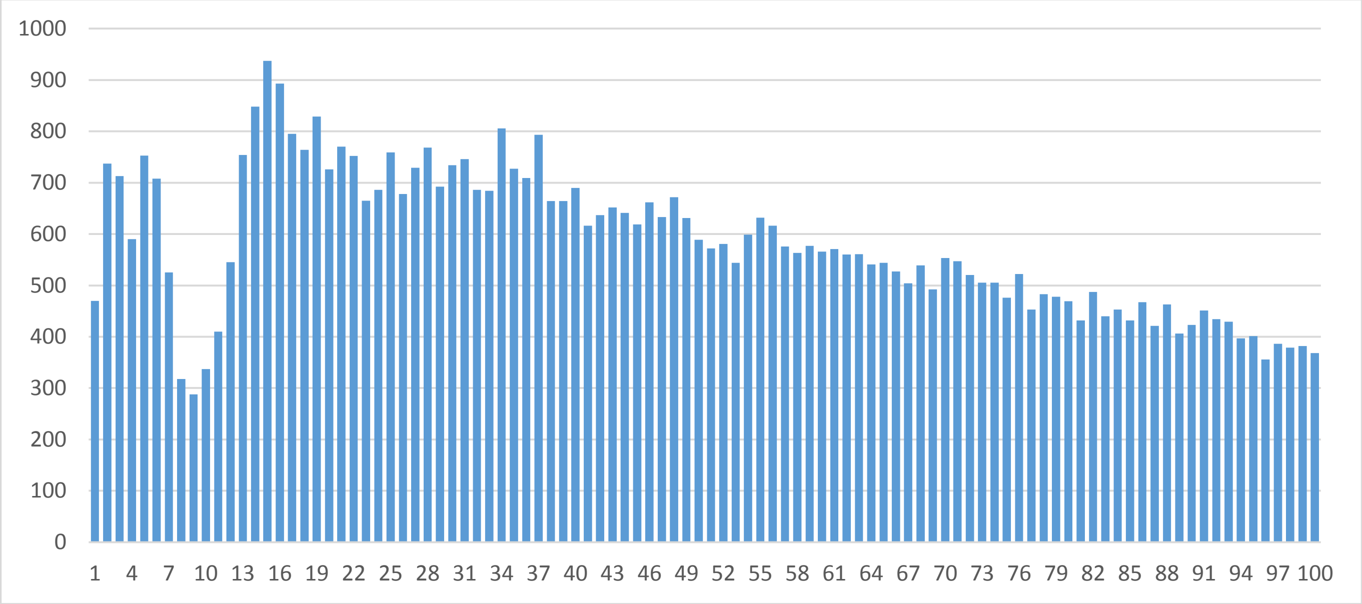

Figure 2.18. All Firmicutes (one genome per genus)

Bacteroides

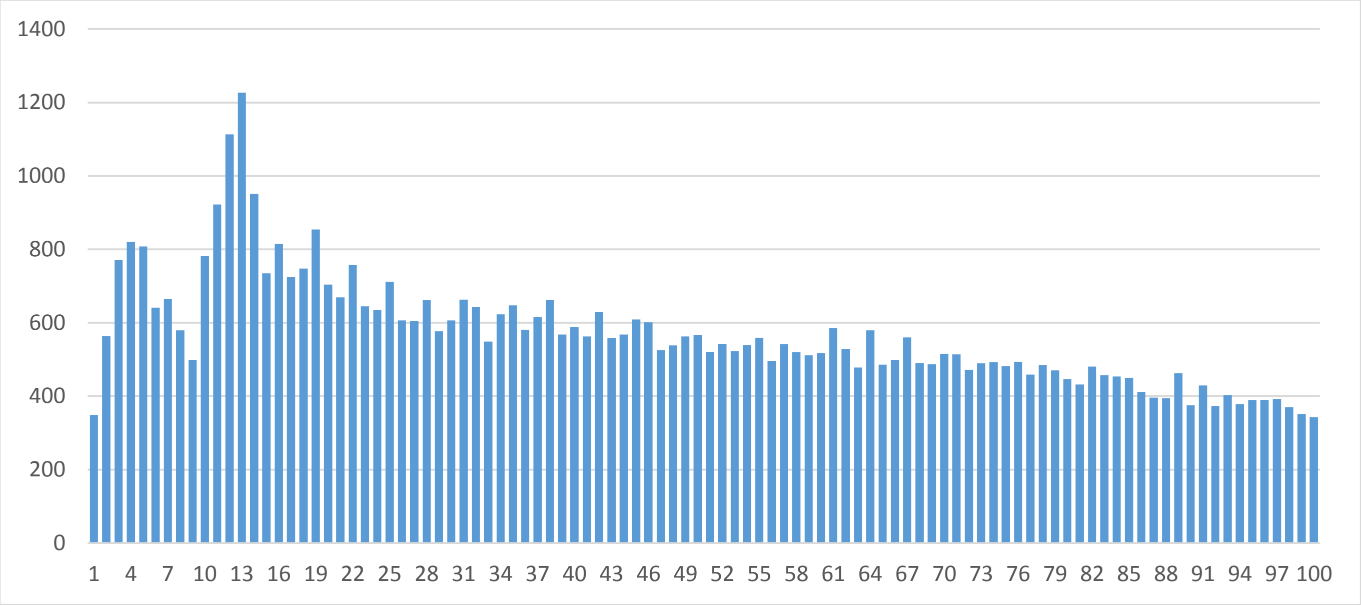

Figure 2.19. All Bacteroides

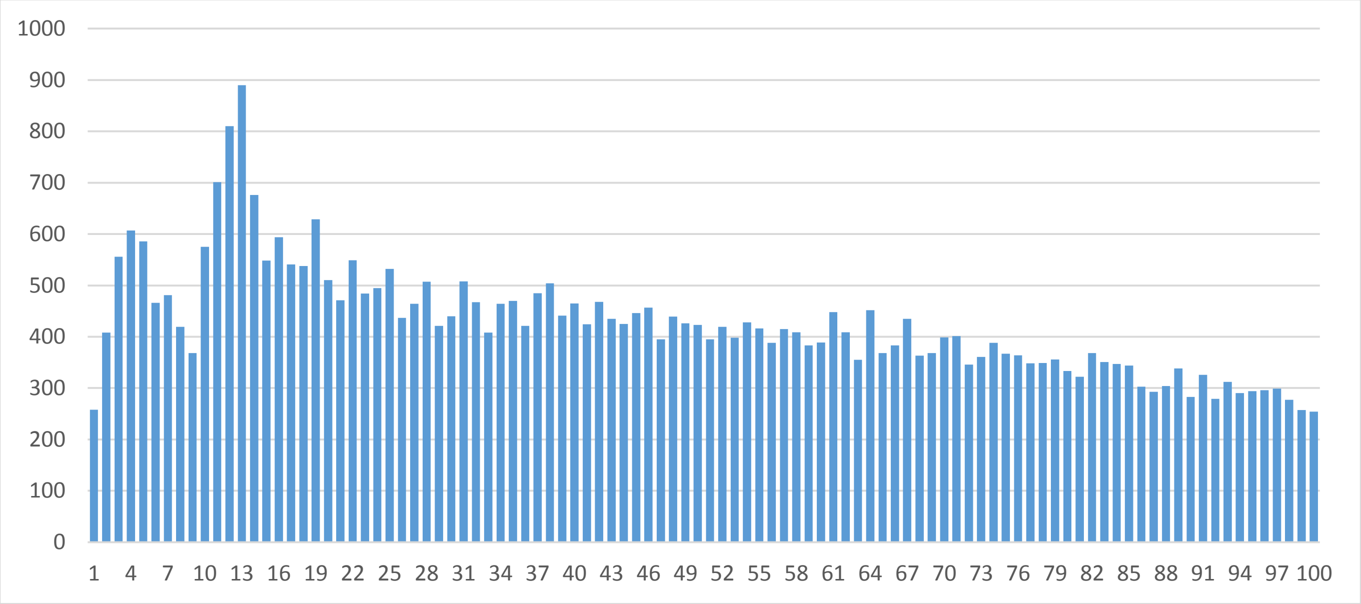

Figure 2.20. All Bacteroides (one genome per genus)

Spirochaetales

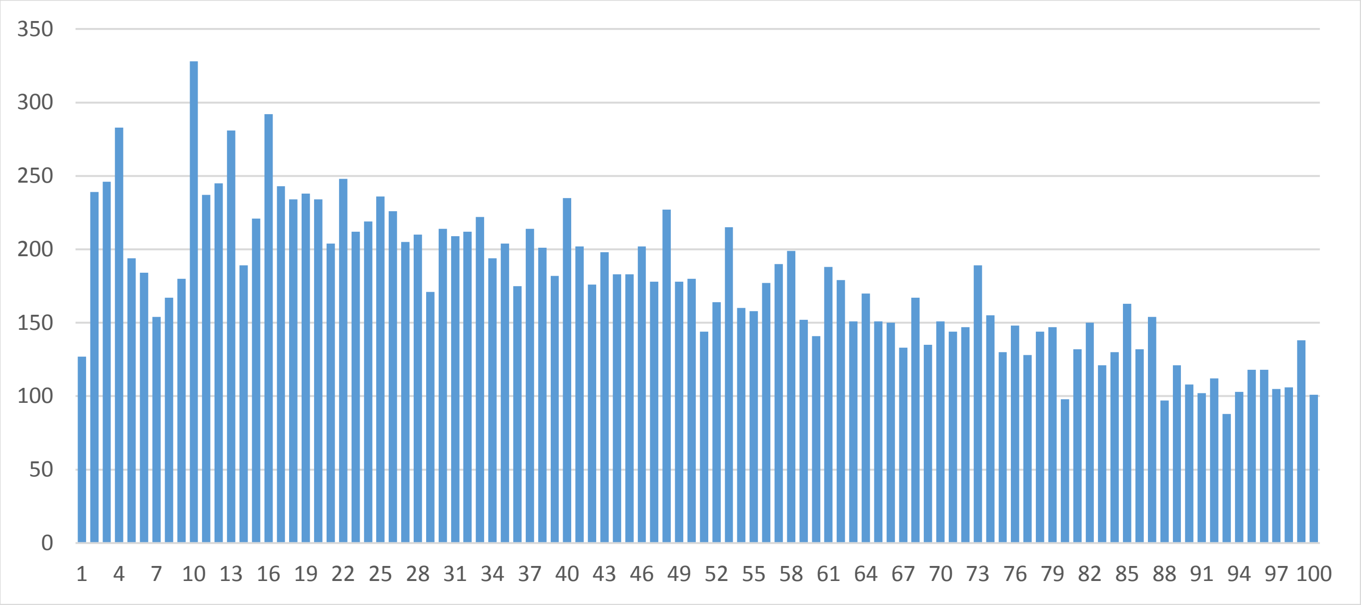

Figure 2.21. All Spirochaetales

Other Bacteria

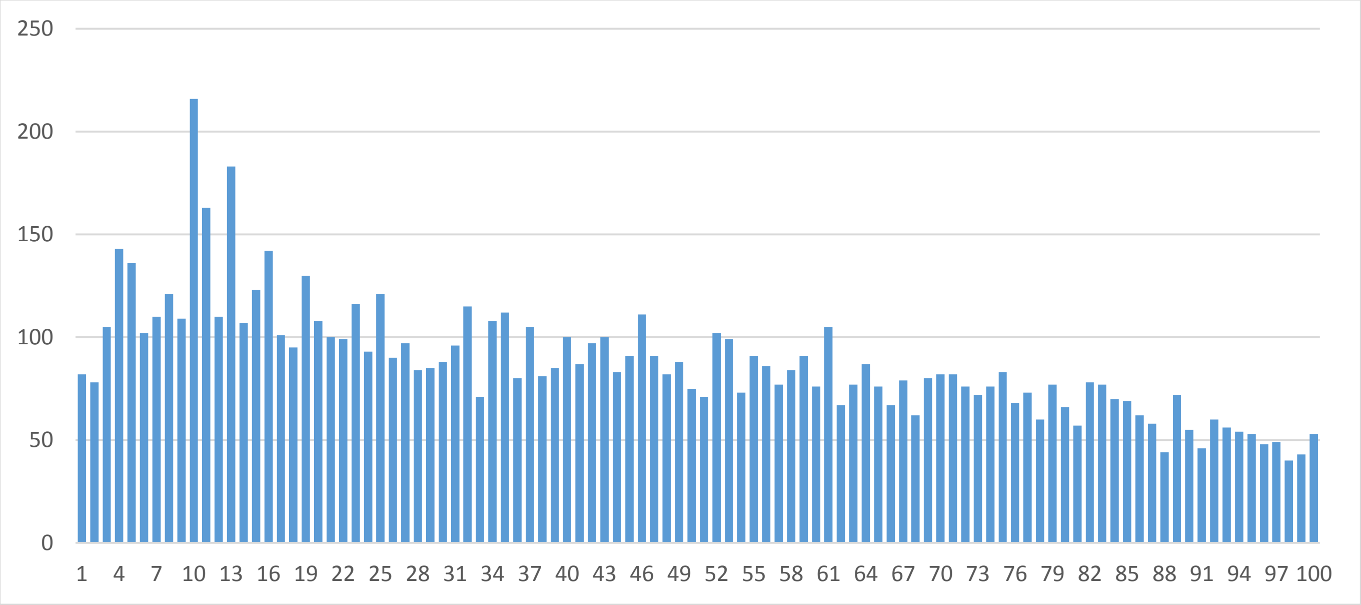

Figure 2.22. Acidobacteria

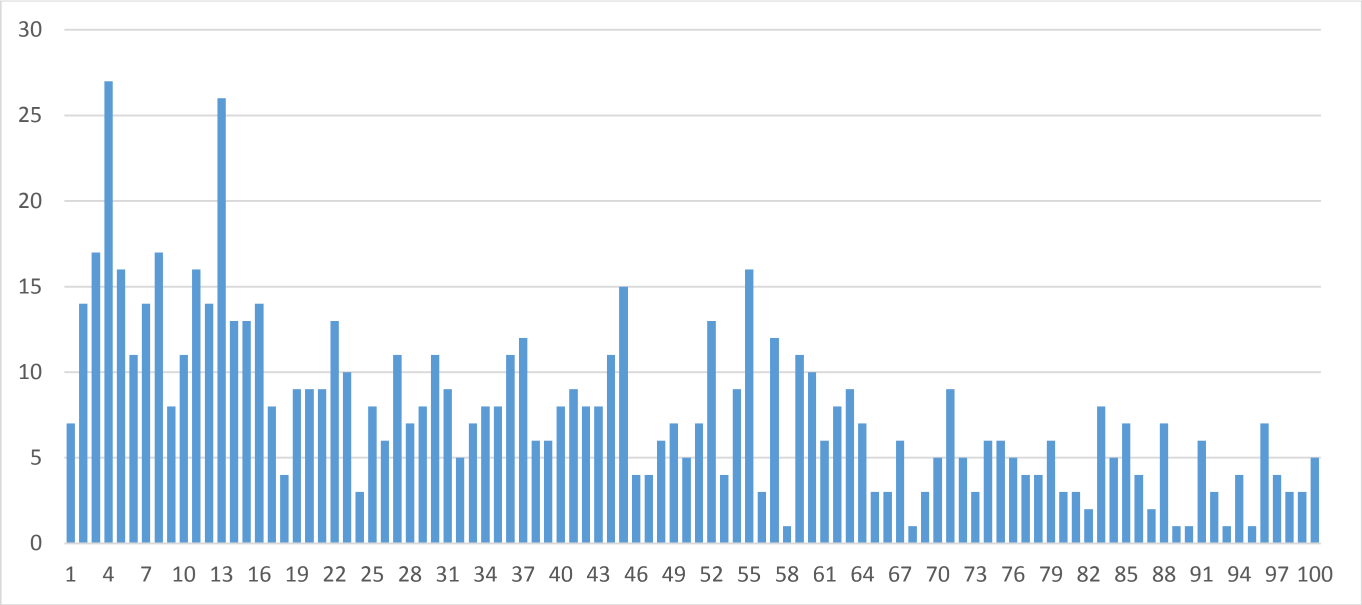

Figure 2.23. Aquificaceae

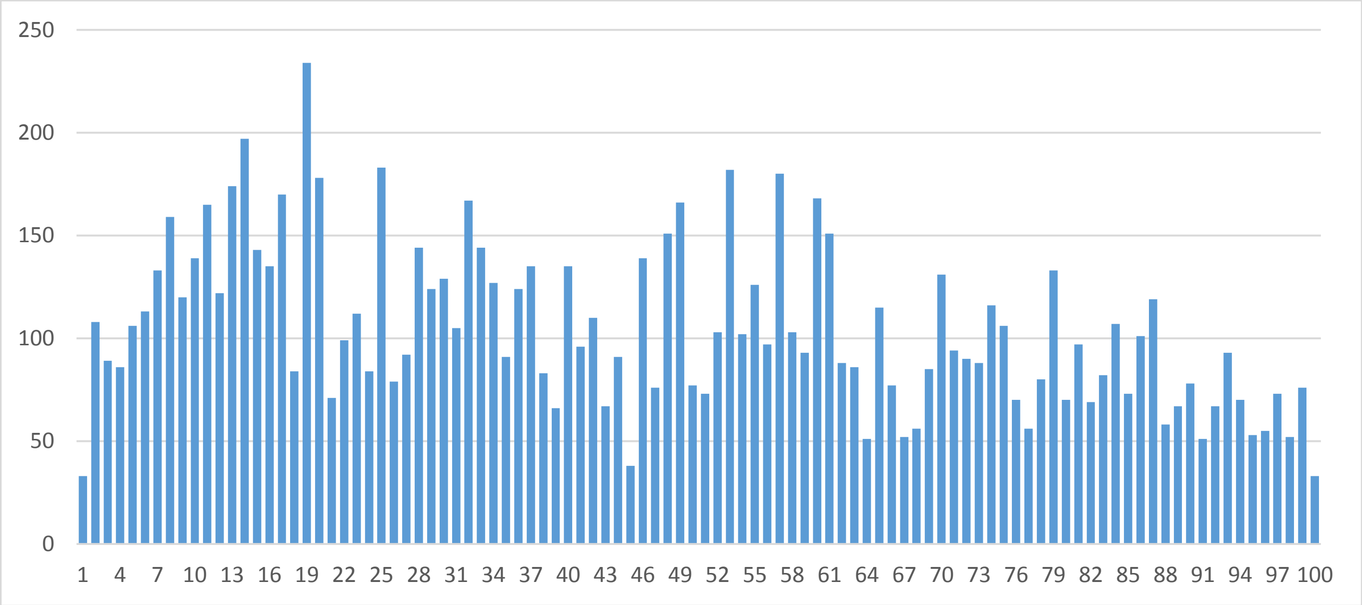

Figure 2.24. Chlamydiae

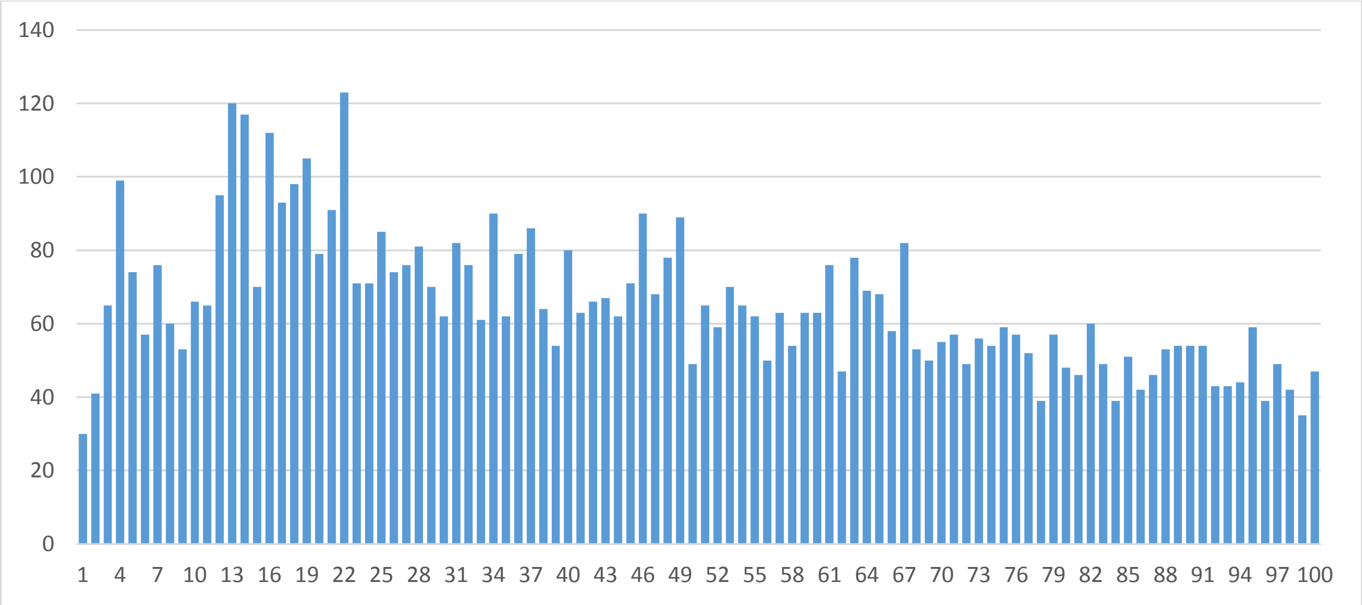

Figure 2.25. Chlorobia

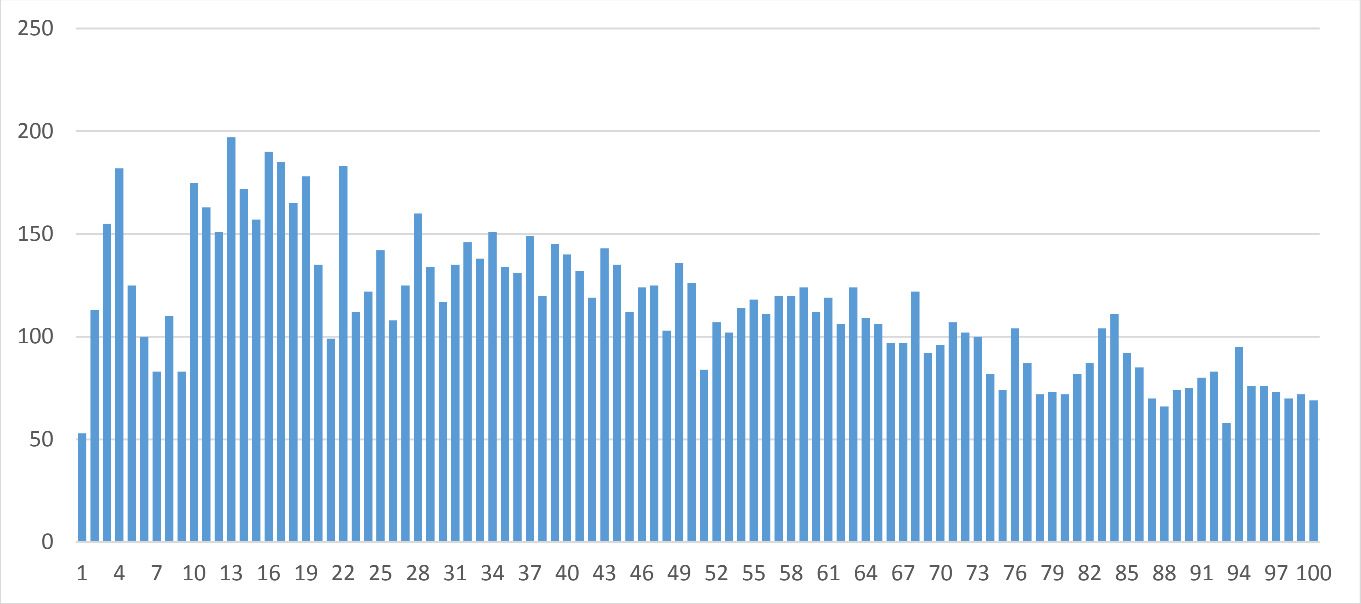

Figure 2.26. Chloroflexi

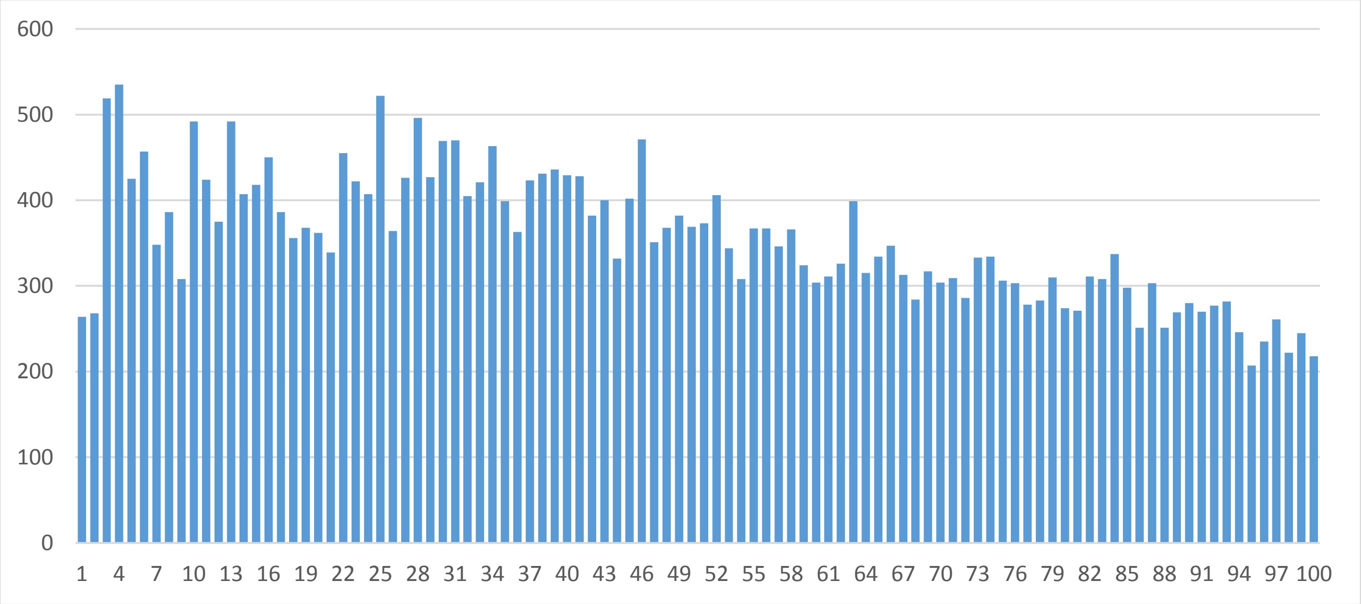

Figure 2.27. Cyanobacteria

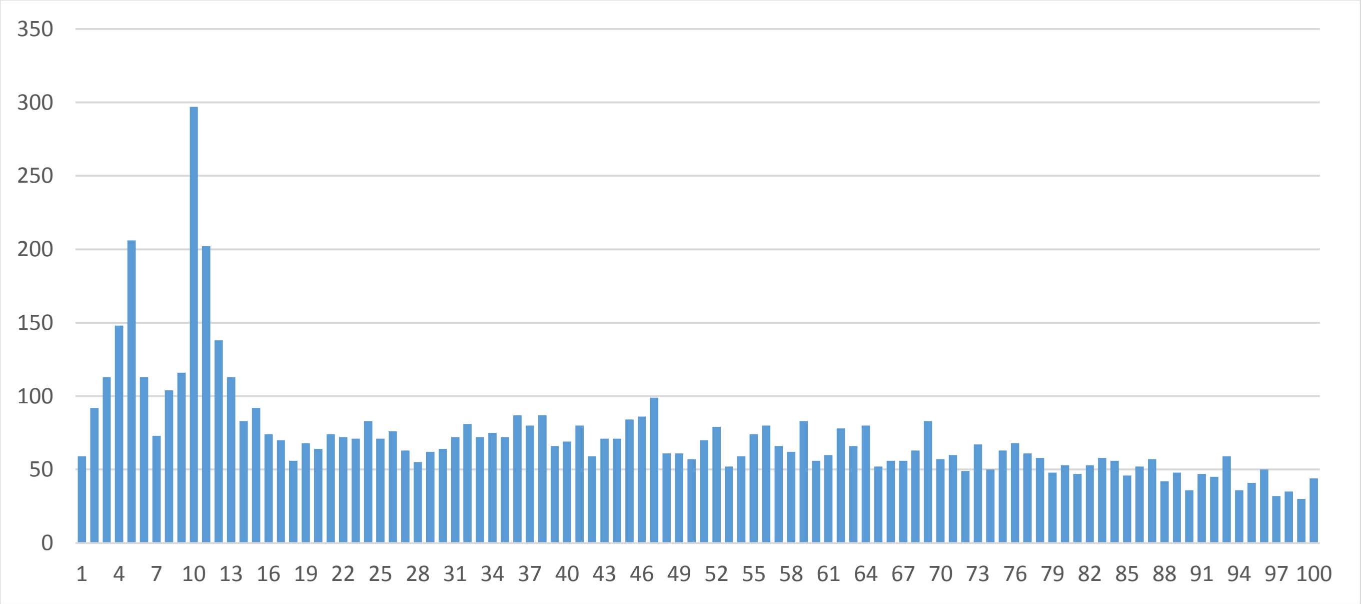

Figure 2.28. Deinococcus-Thermus group

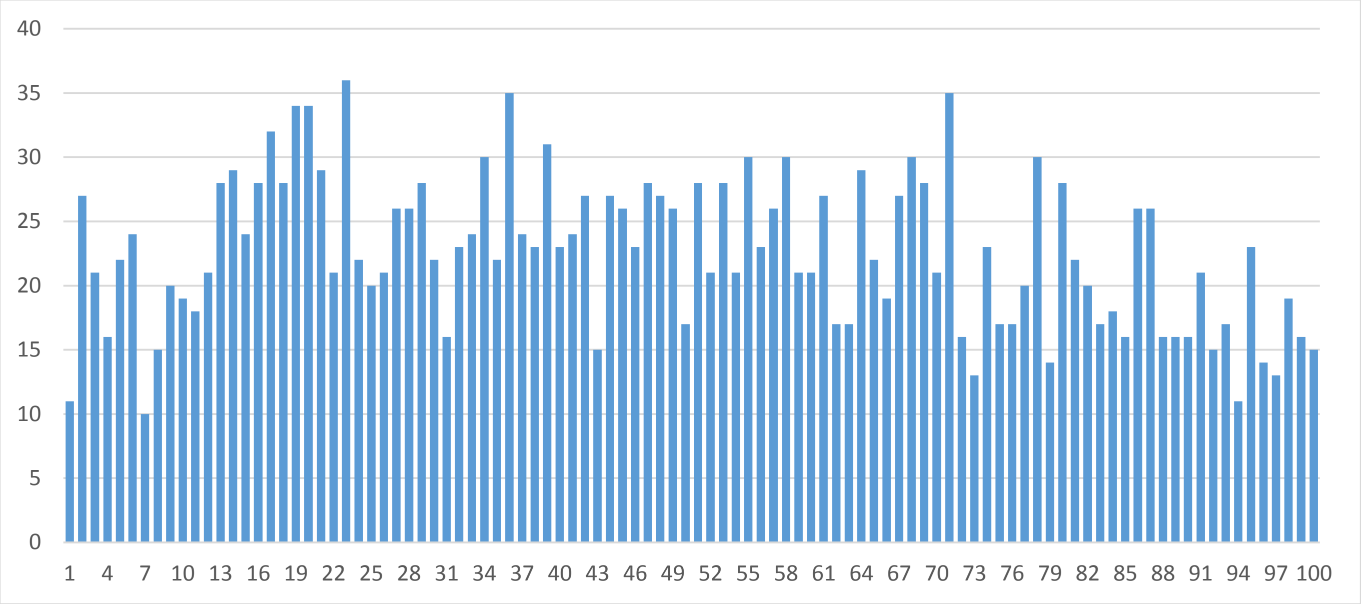

Figure 2.29. Fusobacteria

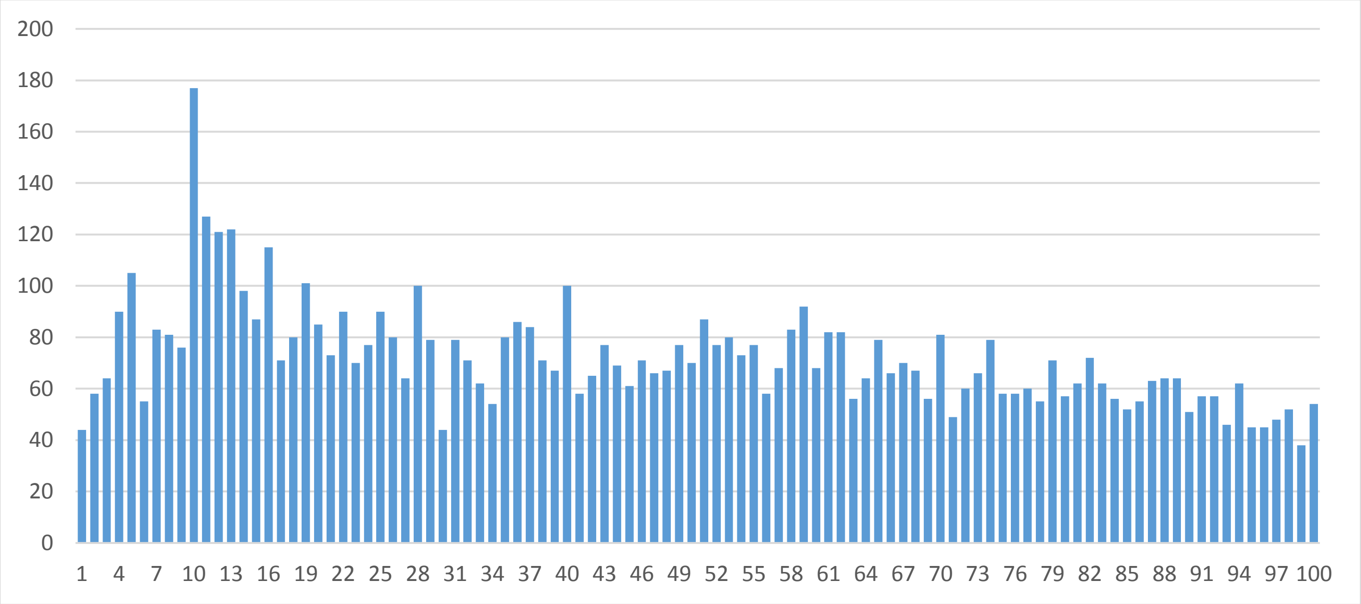

Figure 2.30. Planctomycetes

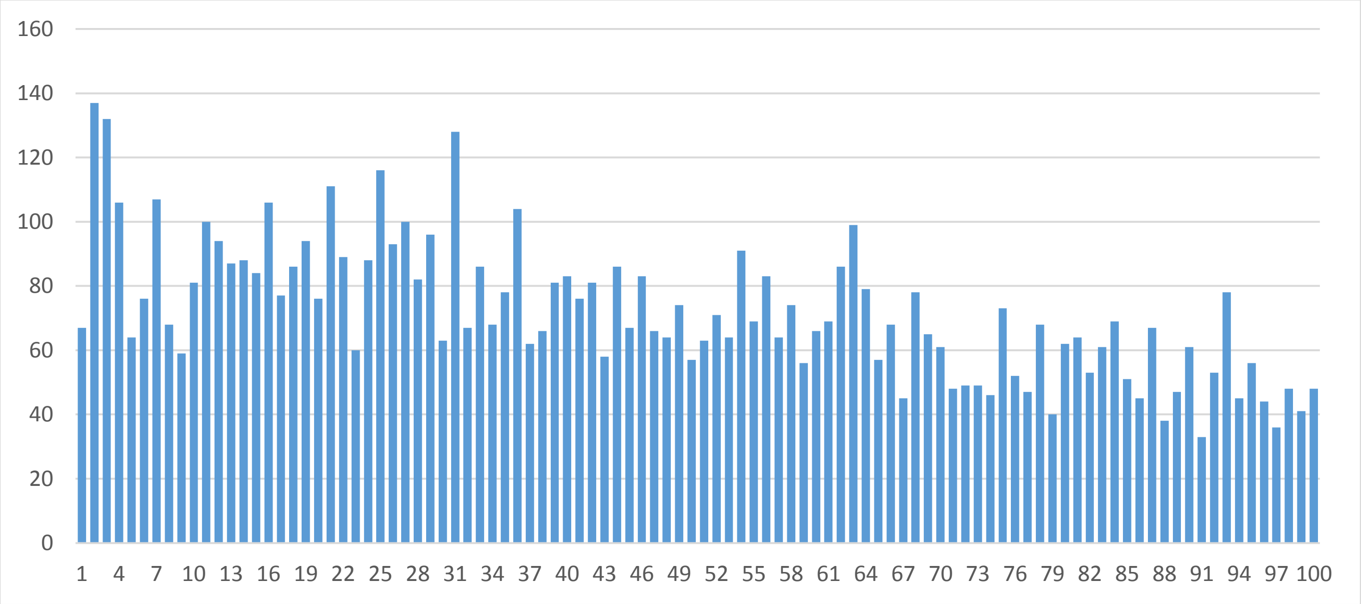

Figure 2.31. Tenericutes

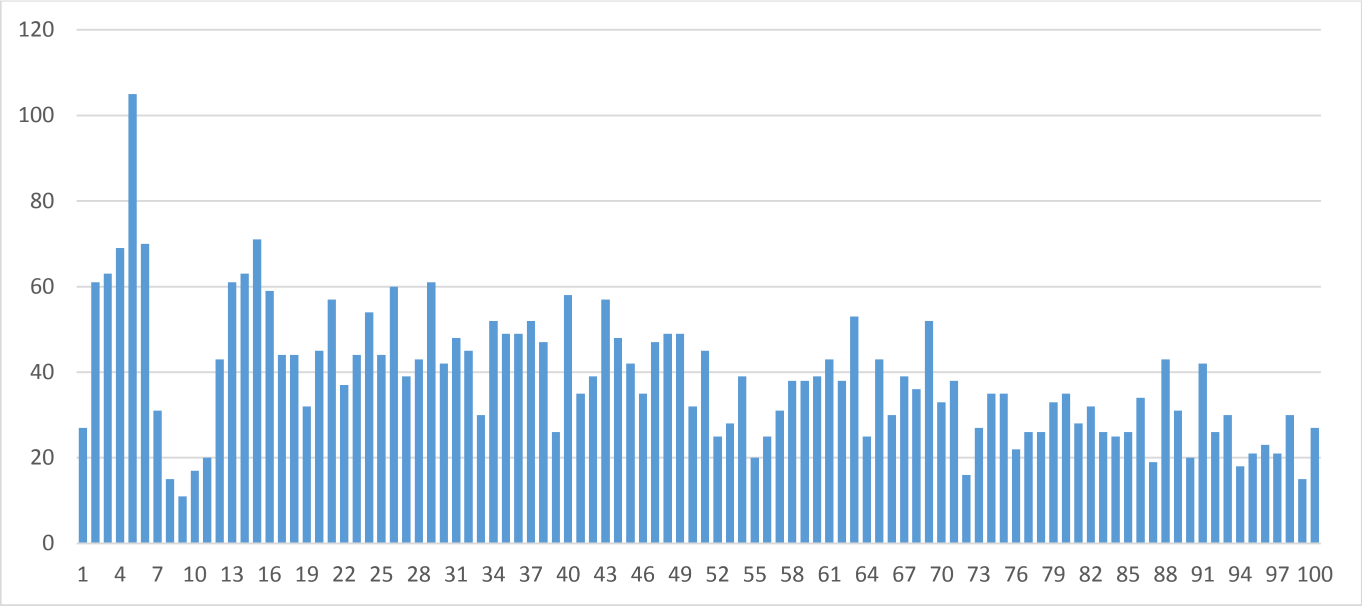

Figure 2.32. Thermotogae
