# Supplementary material for: Ribosome reinitiation at leader peptides increases translation of bacterial proteins
Source: Biol Direct. 2016 Apr 16;11:20. doi: 10.1186/s13062-016-0123-8 (PMC4833913; doi:10.1186/s13062-016-0123-8)

## Part 1. RNA secondary srtructures of 5'-untranslated regions upstream regions encoding proteins with the domains PF002710 and PF00271 in *Corynebacterium diphtheria, C. glutamicum,* and *Bifidobacterium animalis*

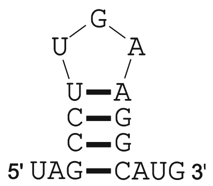


Figure 1.1. RNA duplex in the region from the stop codon of the leader gene to the start codon of the structural gene encoding helicase in *C. diphtheria*

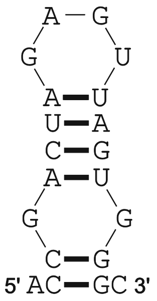


Figure 1.2. RNA hairpin overlapping the Shine-Dalgarno sequence in the helicase in *C. glutamicum*

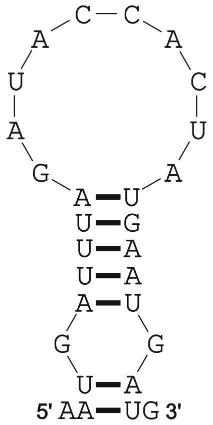


Figure 1.3. RNA hairpin overlapping two nucleotides of the helicase start codon in *B. animalis* and *Streptomyces griseus*

**Part 2. Frequency of the leader-structural gene pairs as a function of the leader gene stop codon in Spirochaetales, Acidobacteria, Deinococcus-Thermus group, and Planctomycetes**

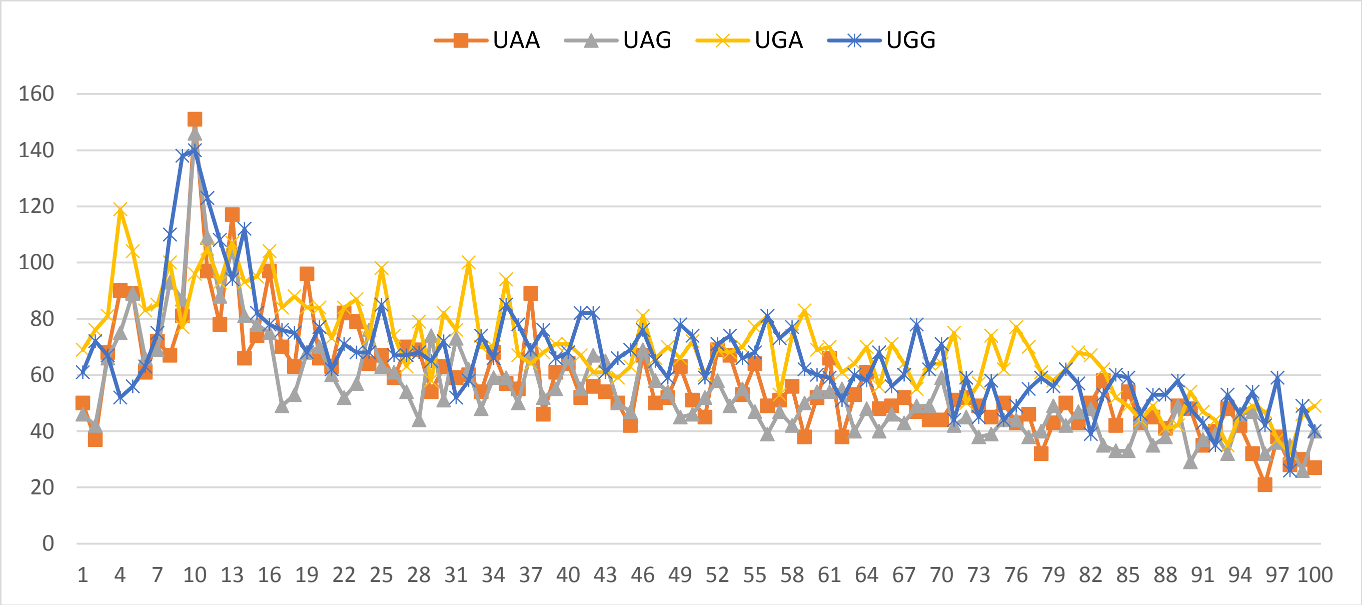


Figure 2.1. Acidobacteria

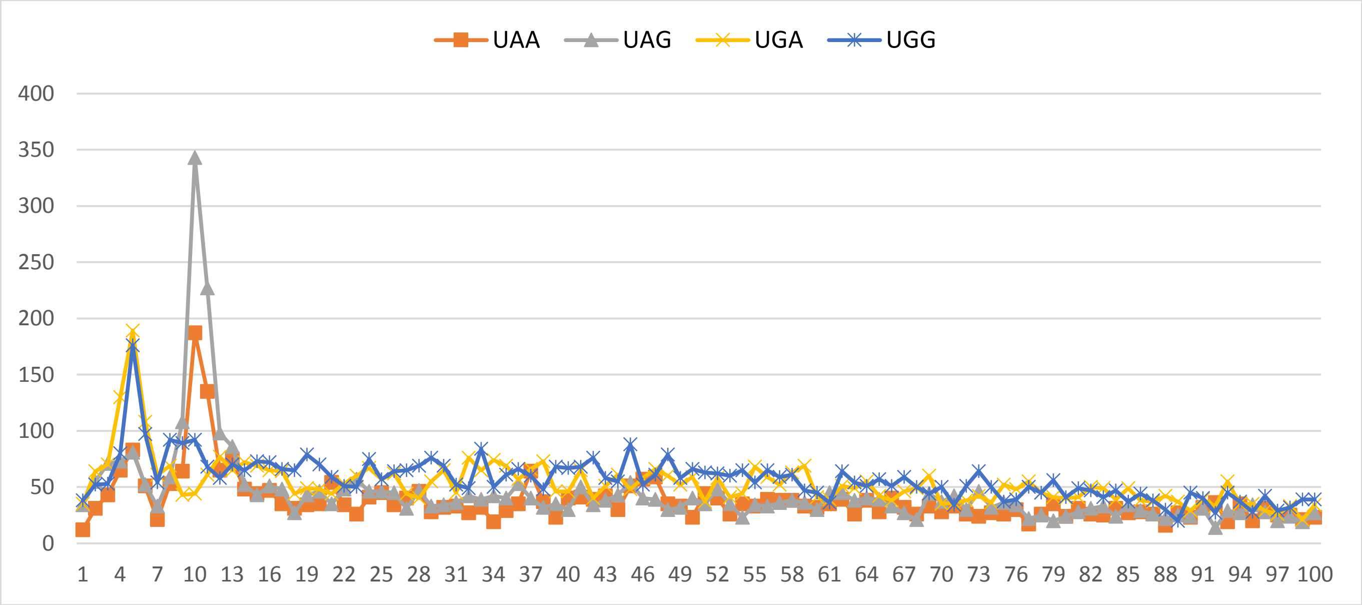


Figure 2.2. Deinococcus-thermus

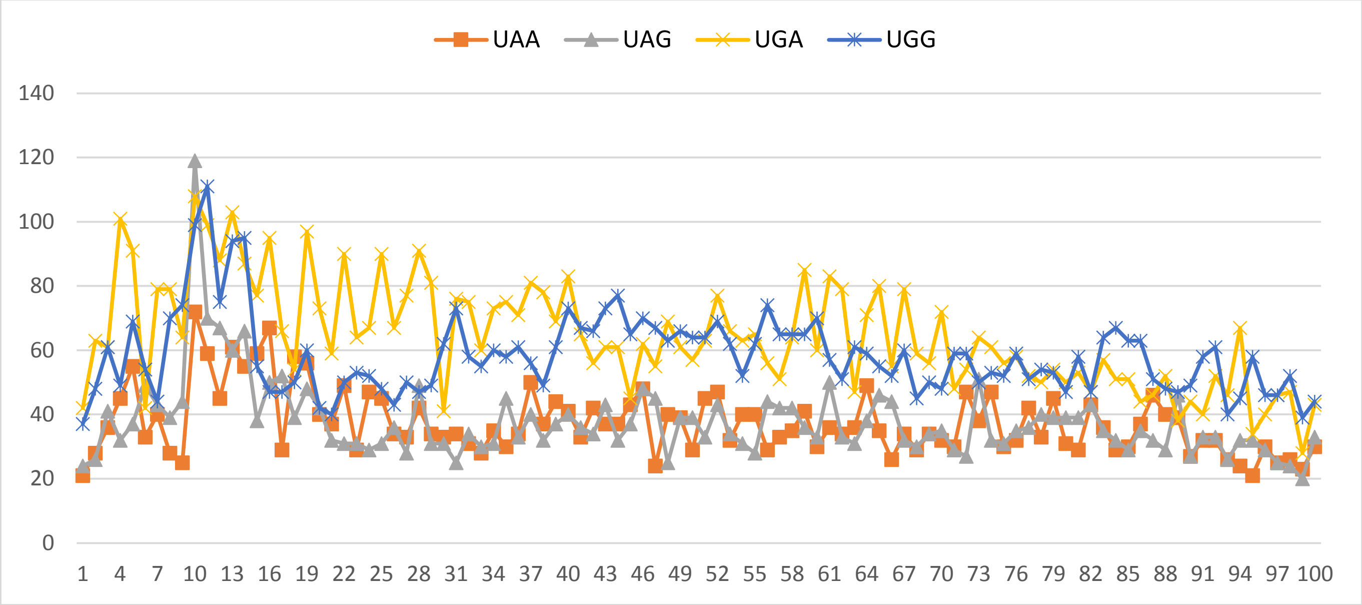

Figure 2.3. Planctomycetes

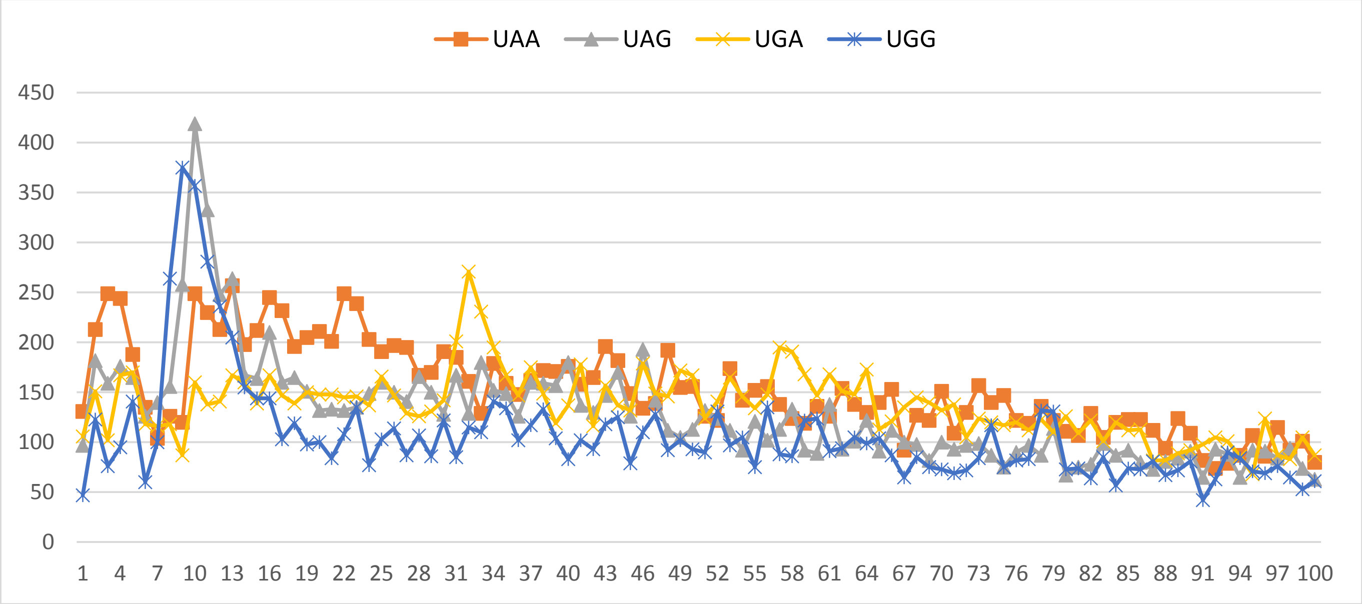

Figure 2.4. Spirochaetales

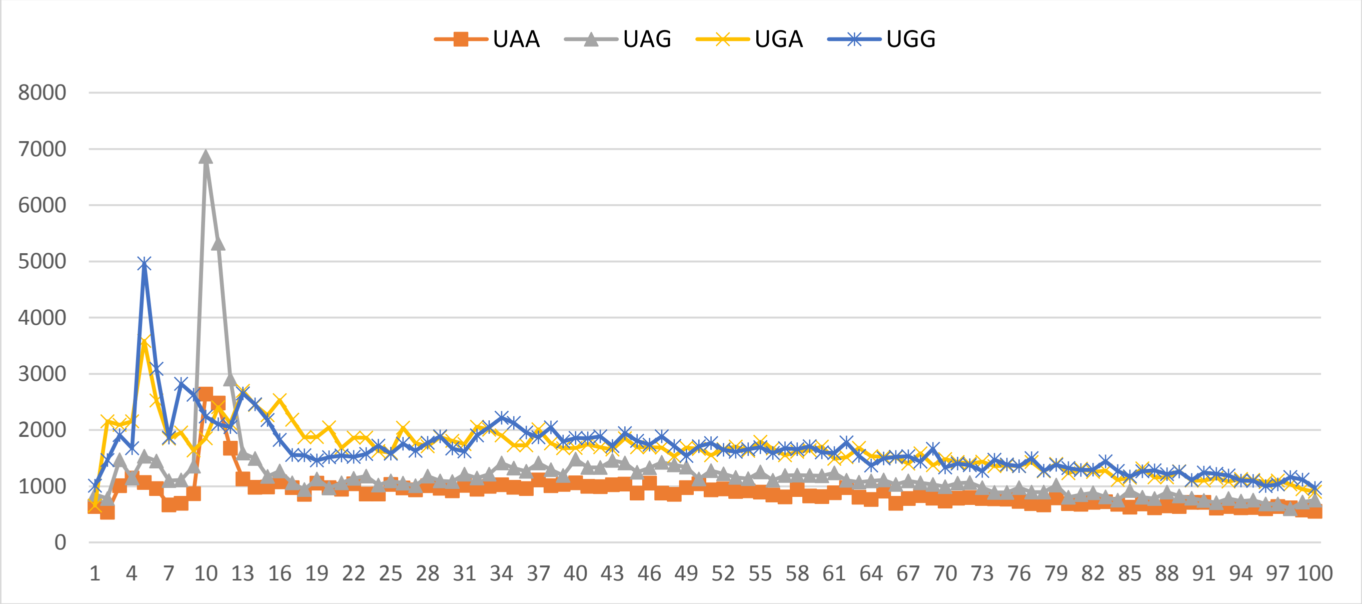

Figure 2.5. Actinobacteria

## Part 3. Sequence logo of the 30-nt 5'-leader regions of all structural genes in Actinobacteria

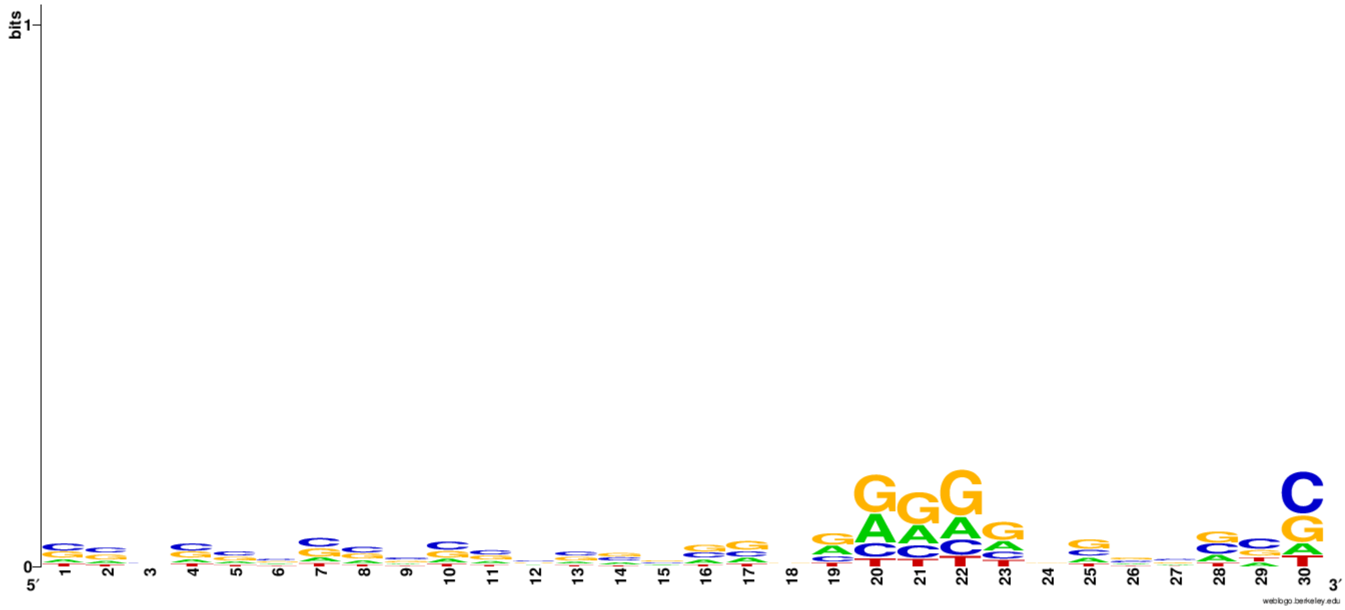

Supplement: Additional file 3: — Part 1. RNA secondary structures of 5'-untranslated regions of proteins with the PF00270 and PF00271 domains in Corynebacterium diphtheria, C. glutamicum, and Bifidobacterium animalis. Figure S1.1. RNA duplex in the region from the stop codon of the leader gene to the start codon of the structural gene encoding helicase in C. diphtheria; Figure S1.2. RNA hairpin overlapping the Shine-Dalgarno sequence in the helicase in C. glutamicum; Figure S1.3. RNA hairpin overlapping two nucleotides of the helicase start codon in B. animalis and Streptomyces griseus. Part 2. Frequency of the leader-structural gene pairs as a function of the leader gene stop codon in Spirochaetales, Acidobacteria, Deinococcus-Thermus group, and Planctomycetes. Figure S2.1. Acidobacteria; Figure S2.2. Deinococcus–Thermus group; Figure S2.3. Planctomycetes; Figure S2.4. Spirochaetales; Figure S2.5. Actinobacteria. Part 3. Sequence logo of the 30-nt 5'-leader regions of all structural genes in Actinobacteria. (PDF 365 kb) [file 13062_2016_123_MOESM3_ESM.pdf]
