# Supplementary material for: Ribosome reinitiation at leader peptides increases translation of bacterial proteins
Source: Biol Direct. 2016 Apr 16;11:20. doi: 10.1186/s13062-016-0123-8 (PMC4833913; doi:10.1186/s13062-016-0123-8)
Supplement: Additional file 4: — Part 1. Frequency plots for the distance between two adjacent structural genes in Escherichia coli K-12, Bacillus subtilis 168 and Synechocystis sp. PCC 6803. Part 2. Frequency plots for the distance between two adjacent structural genes in Actinobacteria, Cyanobacteria, Firmicutes, Proteobacteria, and Spirochaetales. (PDF 126 kb) [file 13062_2016_123_MOESM4_ESM.pdf]

Part 1. Frequency plots for the distance between two adjacent structural genes in *Escherichia coli* K-12, *Bacillus subtilis* 168 and *Synechocystis* sp. PCC 6803

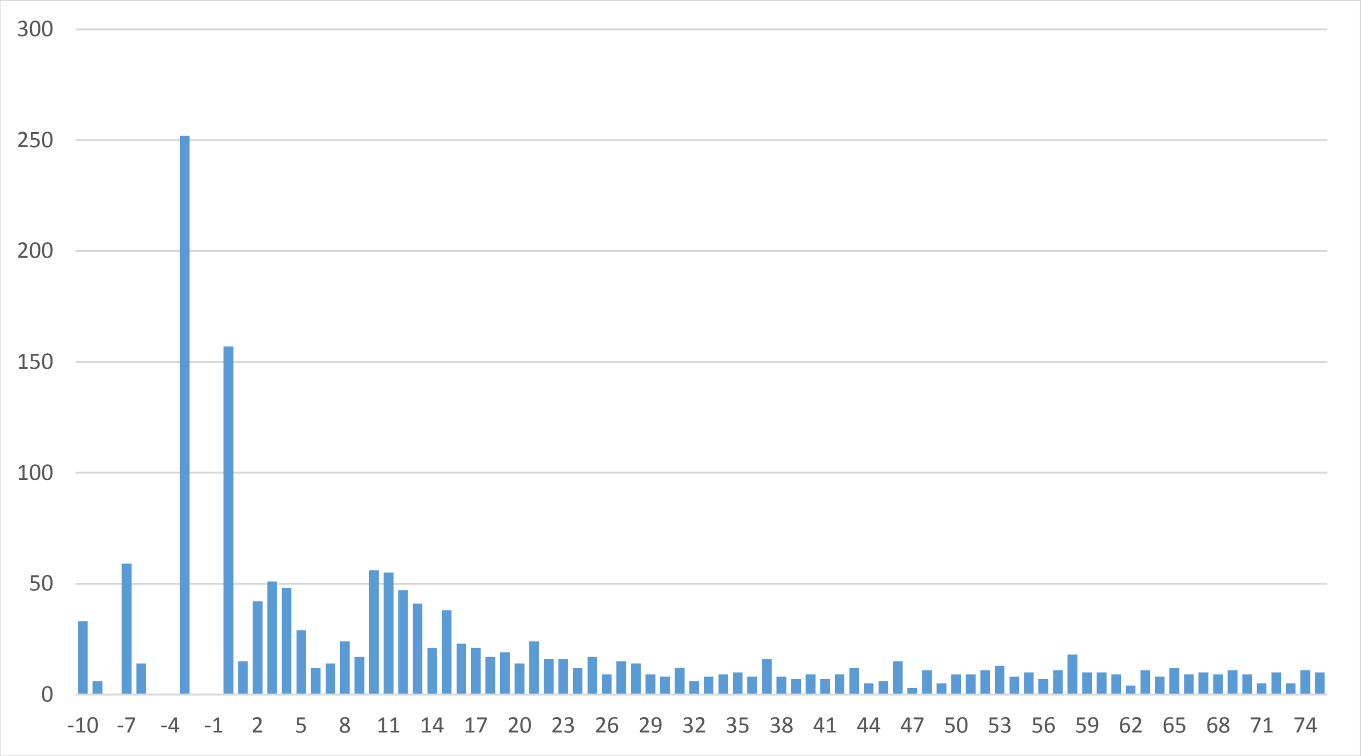

Figure 1.1. *Escherichia coli* K-12

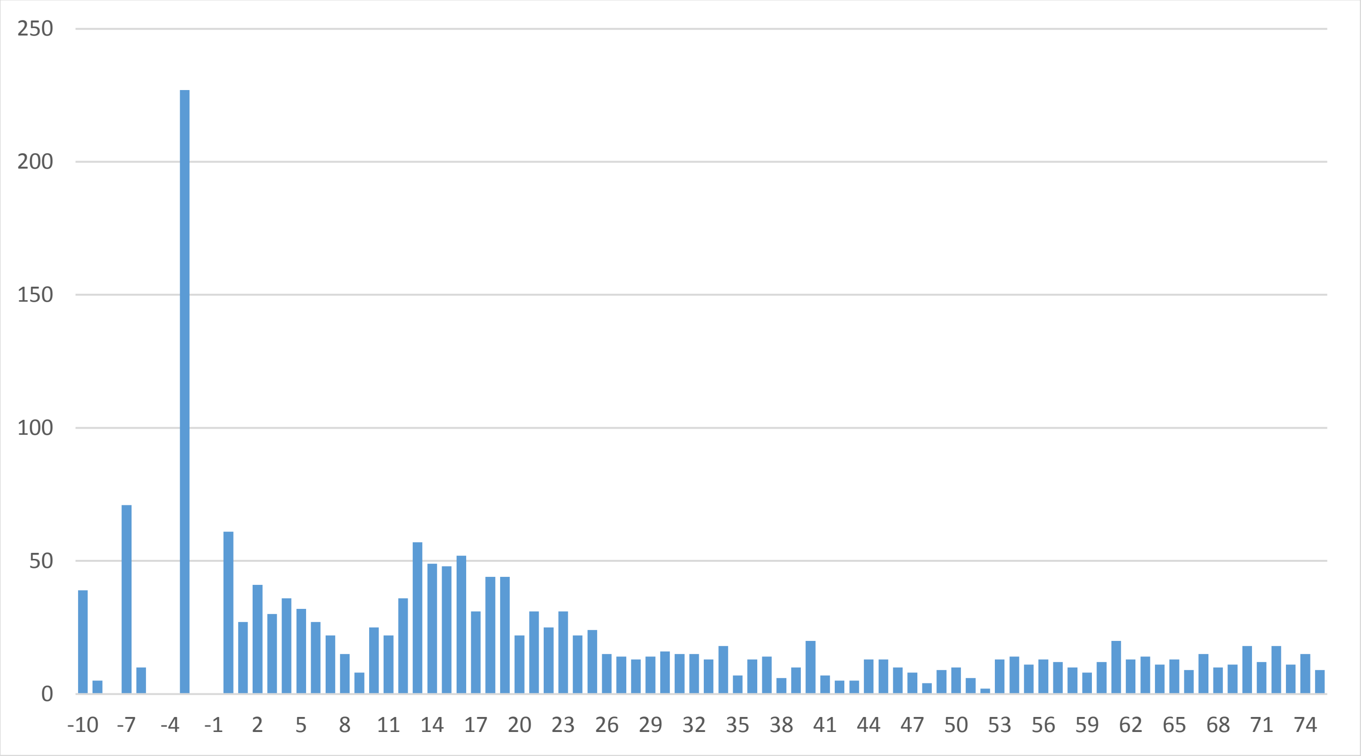

Figure 1.2. *Bacillus subtilis* 168

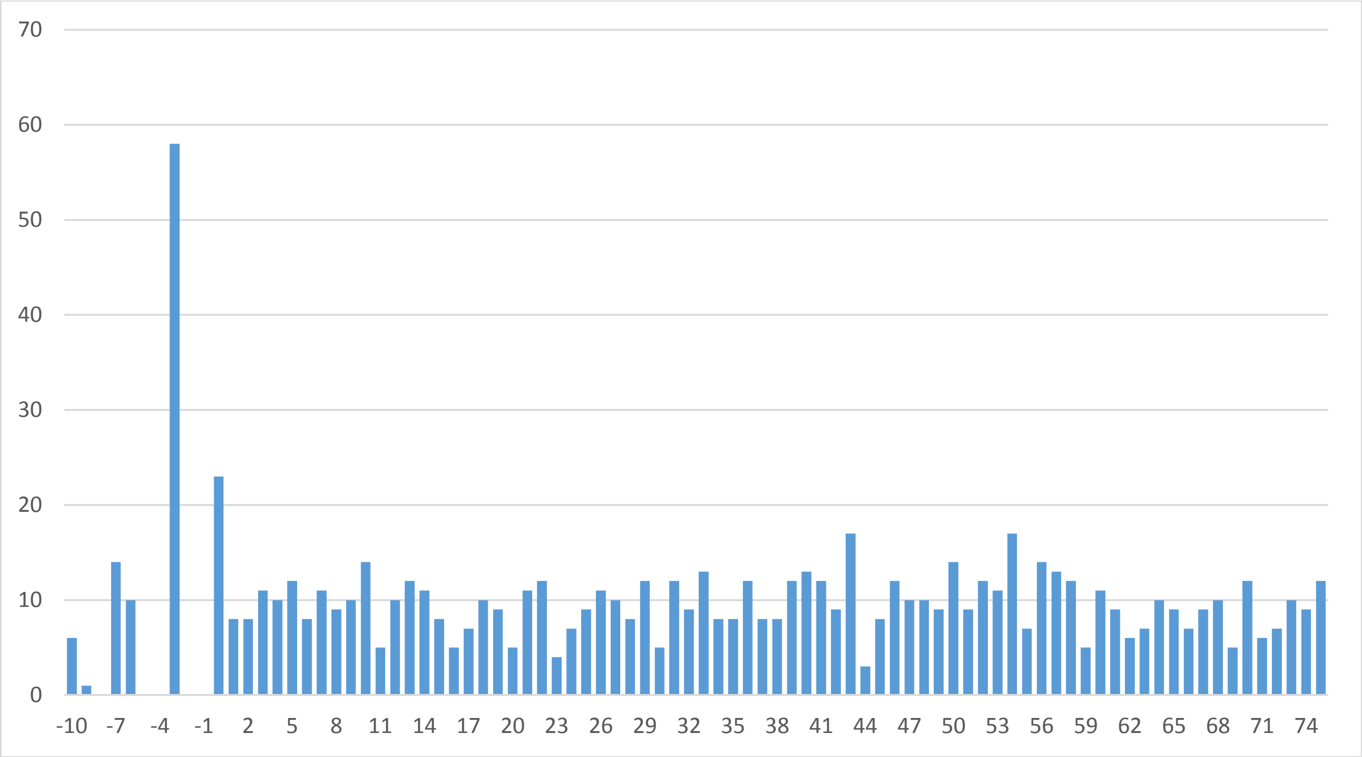

Figure 1.3. *Synechocystis* sp. PCC 6803

Part 2. Frequency plots for the distance between two adjacent structural genes in Actinobacteria, Cyanobacteria, Firmicutes, Proteobacteria, and Spirochaetales

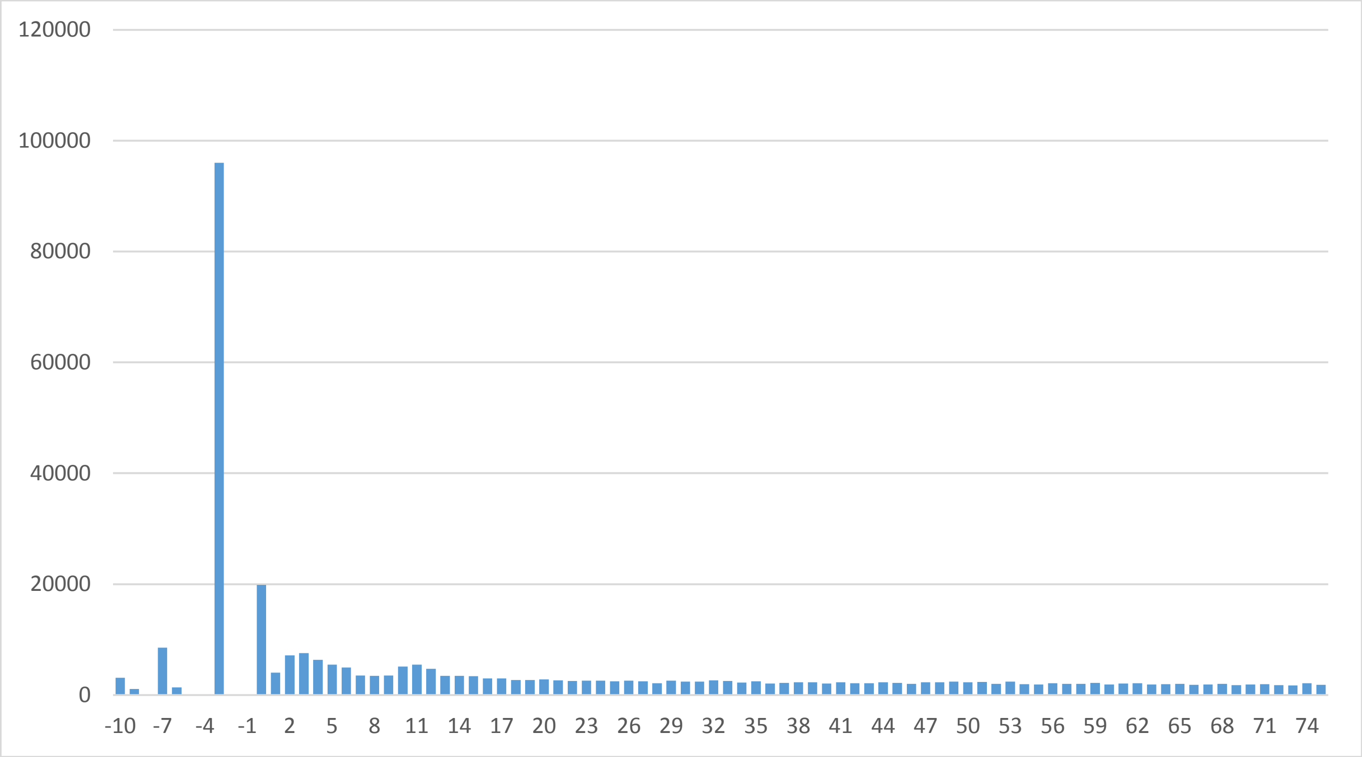

Figure 2.1. Actinobacteria

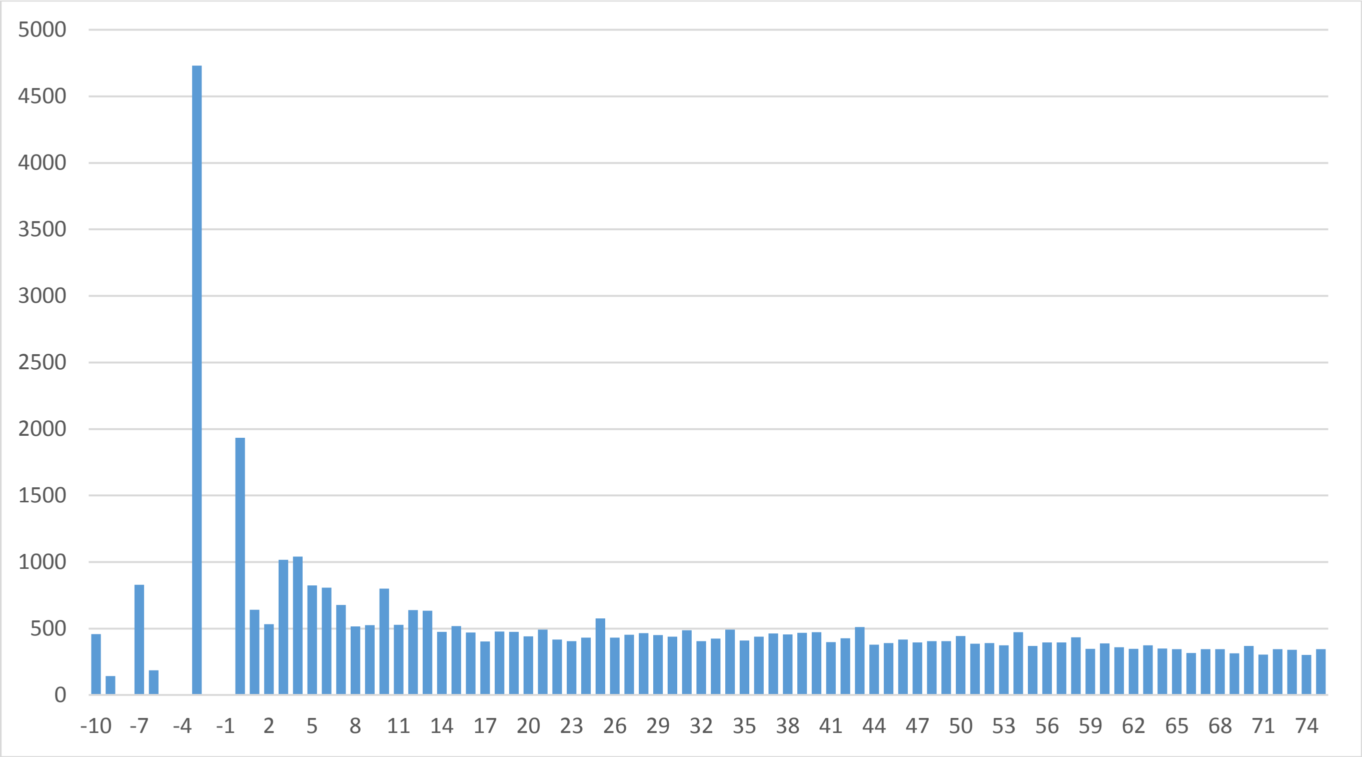

Figure 2.2. Cyanobacteria

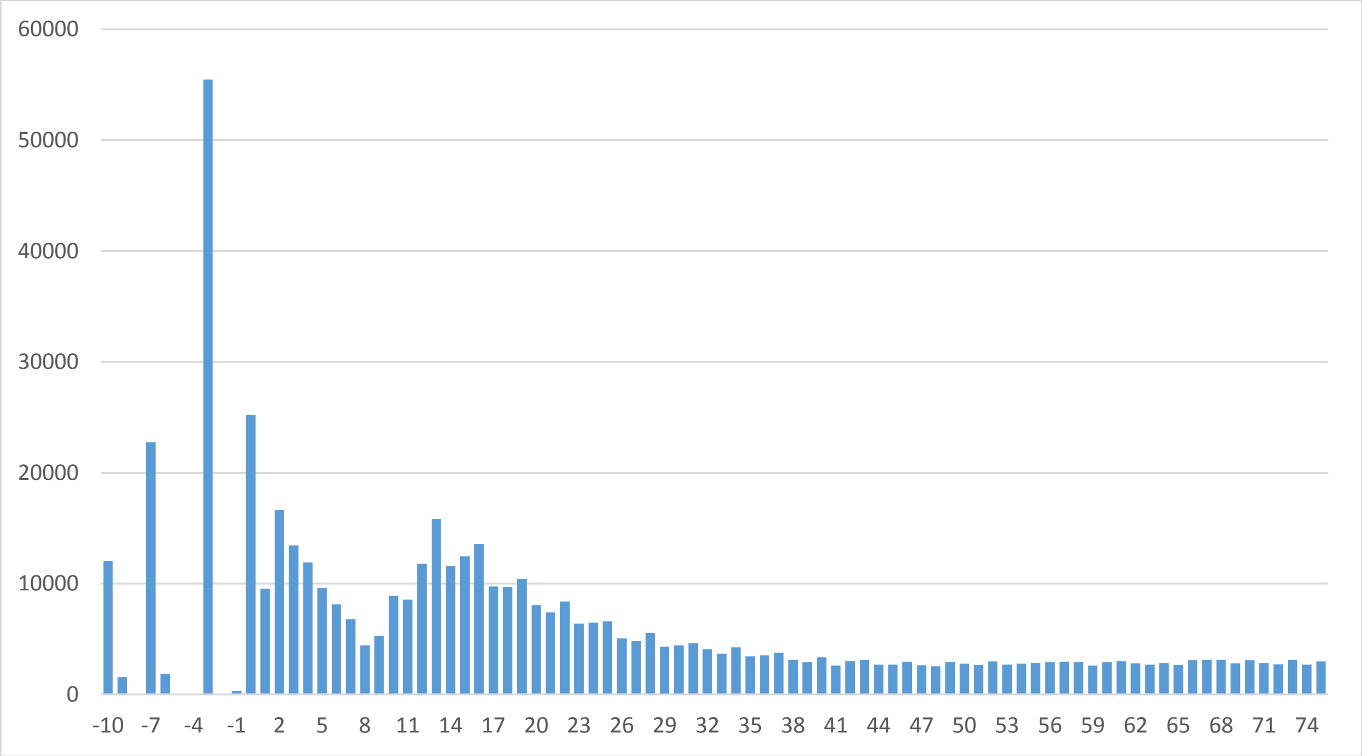

Figure 2.3. Firmicutes

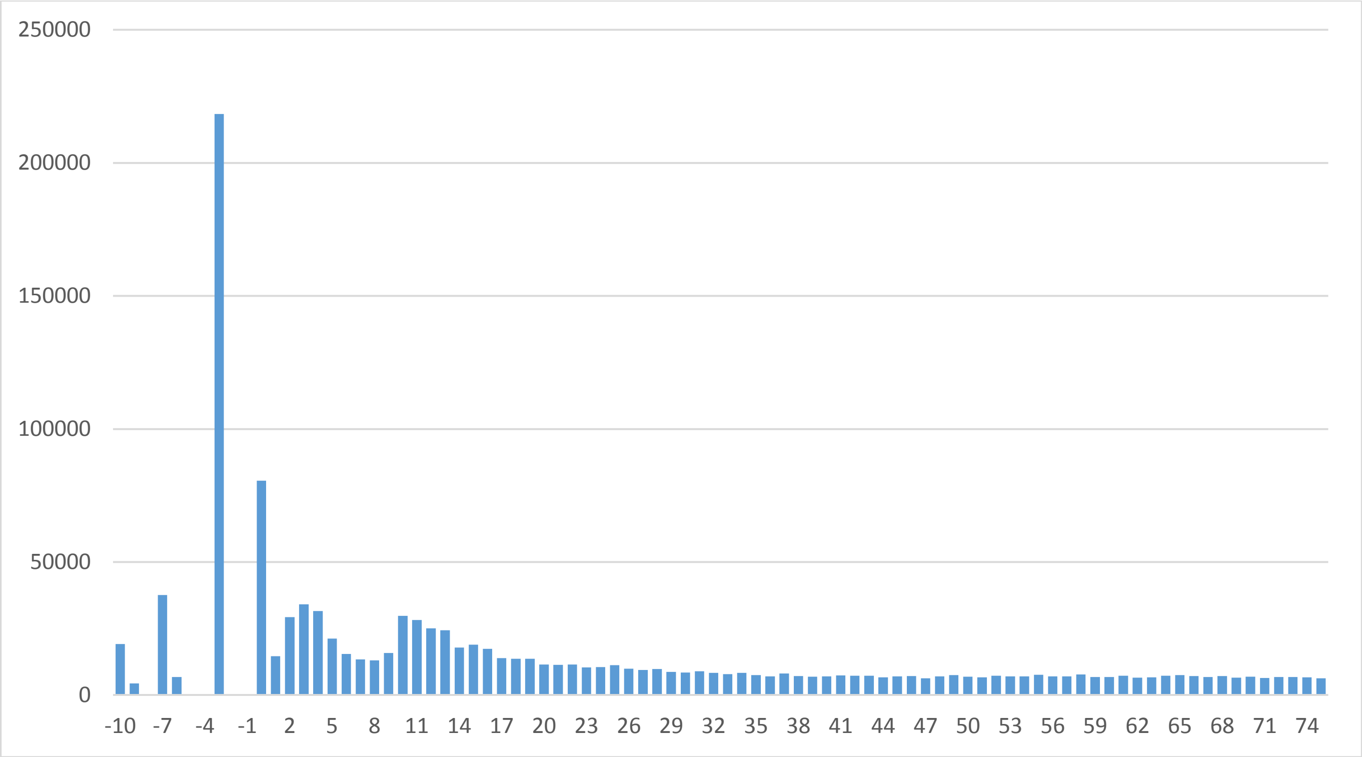

Figure 2.4. Proteobacteria

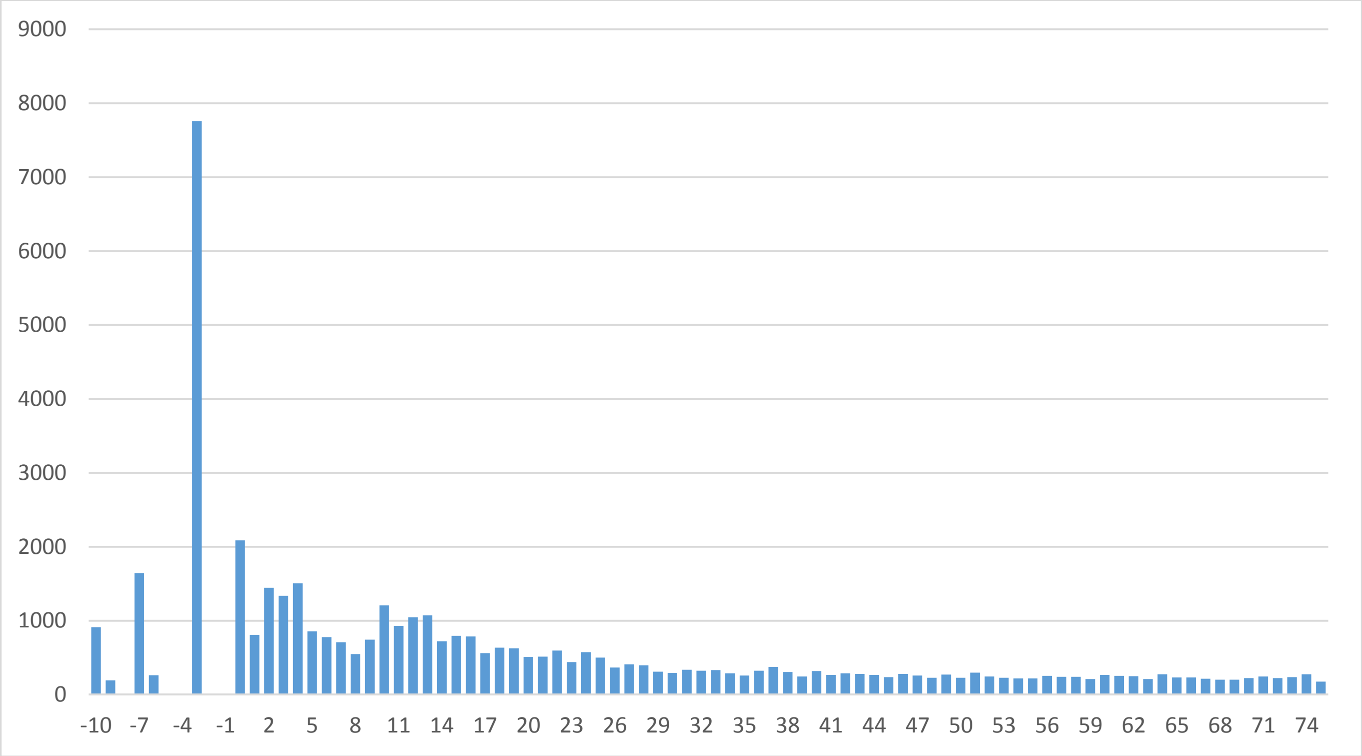

Figure 2.5. Spirochaetales
